# Potassium iodide reduces the stability of triple-cation perovskite solar cells†

**DOI:** 10.1039/d0ra07107b

**Published:** 2020-11-06

**Authors:** Tarek I. Alanazi, Onkar S. Game, Joel A. Smith, Rachel C. Kilbride, Claire Greenland, Rahul Jayaprakash, Kyriacos Georgiou, Nicholas J. Terrill, David G. Lidzey

**Affiliations:** Department of Physics and Astronomy, University of Sheffield Sheffield S3 7RH UK d.g.lidzey@sheffield.ac.uk; Department of Physics, College of Science, Northern Border University Arar 73222 Kingdom of Saudi Arabia; Diamond Light Source Ltd, Harwell Science and Innovation Campus Fermi Ave Didcot Oxfordshire OX11 0DE UK

## Abstract

The addition of alkali metal halides to hybrid perovskite materials can significantly impact their crystallisation and hence their performance when used in solar cell devices. Previous work on the use of potassium iodide (KI) in active layers to passivate defects in triple-cation mixed-halide perovskites has been shown to enhance their luminescence efficiency and reduce current–voltage hysteresis. However, the operational stability of KI passivated perovskite solar cells under ambient conditions remains largely unexplored. By investigating perovskite solar cell performance with SnO_2_ or TiO_2_ electron transport layers (ETL), we propose that defect passivation using KI is highly sensitive to the composition of the perovskite–ETL interface. We reconfirm findings from previous reports that KI preferentially interacts with bromide ions in mixed-halide perovskites, and – at concentrations >5 mol% in the precursor solution – modifies the primary absorber composition as well as leading to the phase segregation of an undesirable secondary non-perovskite phase (KBr) at high KI concentration. Importantly, by studying both material and device stability under continuous illumination and bias under ambient/high-humidity conditions, we show that this secondary phase becomes a favourable degradation product, and that devices incorporating KI have reduced stability.

## Abstract 2

The addition of alkali metal halides to hybrid perovskite materials can significantly impact their crystallisation and hence their performance when used in solar cell devices.

## Graphical abstract

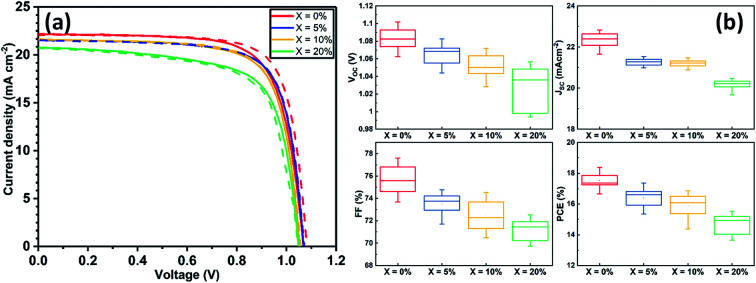

## Introduction

Solution-processed lead halide organic–inorganic perovskites (LHPs) are of significant interest for optoelectronic devices including photovoltaics, light-emitting diodes and radiation sensors.^1–5^ The success of this class of materials has resulted from their high absorption coefficients, low exciton binding energies, long carrier diffusion lengths and extended carrier lifetimes.^6–9^ Importantly, LHPs demonstrate such properties in films deposited from solution that can be been processed at temperatures (80–150 °C) that are an order of magnitude lower than those required to obtain similar figures of merit using inorganic semiconductors such as GaAs and Si.^10^ Photovoltaics that first incorporated LHPs were composed of a single cation species. However, such materials commonly exhibit low thermal stability (*e.g.* methylammonium (CH_3_NH_3_^+^ or MA^+^) lead iodide (MAPbI_3_)) or poor structural stability of the pseudo-cubic phase, *e.g.* formamidinium (HC(NH_2_)_2_^+^ or FA^+^) lead iodide (FAPbI_3_).^11^ Consequently, mixed-cation (Cs^+^, MA^+^, FA^+^) and mixed-halide (I^−^, Br^−^) perovskite compositions have been developed to overcome these limitations, and can form thermally stable photo-active black phases at room temperature.^12^ However, such mixed halide compositions suffer from photo-induced halide segregation and other non-radiative loss mechanisms, resulting in luminescence quantum yields that are often significantly lower than 10%.^13,14^ In order to address these issues, various passivation strategies have been explored, such as the introduction of polymers or larger cations into the perovskite layer that can improve both luminescence quantum yields and moisture stability.^15–21^ However, the presence of such macromolecules often impedes charge transport if not managed properly and controlling mixed phase films using larger cations can also compromise device efficiency.

To directly passivate defects in a perovskite, interest has turned to the use of inorganic additives. Saliba *et al.* explored a triple-cation mixed-halide perovskite composition (denoted throughout this paper as ‘TC’) with the addition of rubidium (Rb^+^), which showed enhanced device performance in a photovoltaic device.^22^ Bu *et al.* later used a similar approach with a smaller monovalent alkali metal halide, potassium (K^+^).^23^ Despite the performance enhancement observed, solid-state NMR studies on TCs with Rb^+^ and MAPbI_3_ with the addition of K^+^ demonstrated that such cations are not incorporated into the perovskite lattice, and it was concluded that they underwent segregation, forming secondary phases.^24^ Recently, Abdi-Jalebi *et al.* reported the effectiveness of potassium iodide (KI) as a defect passivating additive in TC perovskites, resulting in materials having a high photoluminescence quantum yield (PLQY) (reportedly up to 66%) and substantially reduced photo-induced halide segregation.^25^ Indeed, it was proposed that the excess iodide species from KI passivated halide vacancies in the perovskite, while a potassium halide phase resided at the grain boundary region and mitigated photo-induced halide segregation.^25^

Despite such very promising findings, there are still questions regarding the origin of the beneficial effects of alkali metal passivation in TCs (for example whether they result from interface or bulk passivation) and whether such additives confer long-term operational stability upon TC photovoltaic devices. Notably, most of the previous reports on KI passivated perovskite solar cells only investigate their shelf life or operational stability under inert environment (see Table S1 ESI†). However, device stability under real world conditions (*i.e.* continuous illumination, RH >30%, temperature >30 °C) remains unexplored and this forms the basis of our work.

In this manuscript, we first compare the effects of the addition of KI addition on the optical and microstructural properties of TC and confirm previous findings in the reported literature.^23,25–27^ We then perform focussed investigations into the effect of the addition of KI on material and device stability under ambient/high-humidity conditions. Specifically we add KI into a TC precursor solution at different concentrations and fabricate planar perovskite solar cells, comparing the performance of devices using electron transport layers (ETL) formed from a nanoparticle tin oxide (SnO_2_) solution with those based on titanium dioxide (TiO_2_**)**. Our study follows previous work by Abdi-Jalebi *et al.* who observed a monotonic increase in PLQY of TC perovskite on addition of KI at a concentration of up to 40% in the precursor solution, with an optimum performance of PV devices determined at a KI concentration of 10% KI.^25^ Indeed, we have based our experimental methodology on such previous work and have explored a range of KI concentrations in the TC precursor solution, including 0, 5, 10, and 20 mol%. To understand the effect of the KI addition over this concentration range we use a range of characterisation techniques to understand the influence of KI on the structural, morphological and optical properties of the resultant TC films. We show that for KI additions greater than 5 mol%, phase segregation occurs forming an undesirable secondary non-perovskite phase (KBr). We then perform detailed studies on devices that incorporate either a SnO_2_ or TiO_2_ ETL that explores the effect of KI on modifying their operational stability. We show that the addition of KI has a negative effect on PV performance in devices constructed that use a SnO_2_ ETL, however devices based on a TiO_2_ ETL exhibited improved efficiency at KI concentration of 10%. To explain our findings, we propose that residual potassium hydroxide (KOH) that is used as a stabilising agent in the SnO_2_ colloidal solution already partially passivates this interface. This negates the beneficial effects of the KI, despite resulting in some improvement in device hysteresis. Significantly, we show that the presence of the KI additive is correlated with reduced stability of devices incorporating both SnO_2_ and TiO_2_ ETLs. Our study suggests that the passivating effects of KI in TC perovskites occur predominantly at the ETL–perovskite interface and is determined by the degree to which the interface is already passivated.

## Results and discussion

We have investigated the influence of potassium iodide (KI) addition (0–20%) on the photovoltaic performance and stability of a triple-cation mixed-halide perovskite with approximate solution composition Cs_0.05_FA_0.79_MA_0.15_PbI_2.45_Br_0.55_ with an excess of PbI_2_. Henceforth, we refer to the amount of KI added as the molar fraction (in %) of all cations present in the precursor solution, defined^21^ as X = [K]/([K]+[A]) where A = (Cs, FA, MA). In all cases, the composition and concentrations discussed in the paper refer to the concentration of ions in the precursor solution-phase, and we acknowledge that the final composition of such species in the final perovskite film is likely to be slightly different from this due to volatilization during film processing. Furthermore, we emphasize that the concentration range explored here is closely based upon previous work in which a KI concentration of 10% was shown to result in optimum device efficiency.^25,28^

### PV device studies using an SnO_2_ ETL

Devices were fabricated using an n–i–p architecture using a commercially available SnO_2_ nanoparticle solution to fabricate ETLs and spiro-OMeTAD as a hole transport layer (HTL). The structure of the resultant glass/ITO/SnO_2_/perovskite/spiro-OMeTAD/Au device is shown schematically in Fig. S1.† Details of the fabrication processes used are described in ESI.†

**Fig. 1:**
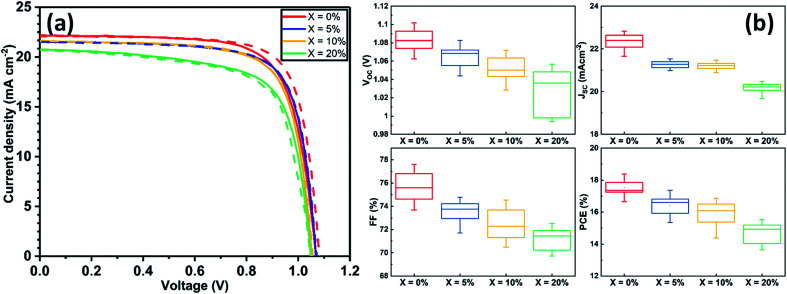
(a) Current density–voltage curves for champion devices incorporating the addition of 0, 5, 10 and 20% KI. Forward (*J*_sc_ to *V*_oc_) and reverse (*V*_oc_ to *J*_sc_) sweep directions are indicated by solid and dashed lines respectively. (b) Statistical box plots for PSCs performance (reverse scan *V*_oc_ to *J*_sc_) determined from all devices.

Current density–voltage (*J*–*V*) performance for the best devices is shown in Fig. 1(a), with Table S2† tabulating champion device metrics (with average values shown in parenthesis) following the addition of 0, 5, 10 and 20% KI. Notably, the best performing control TC device (having 0% KI) had the highest power conversion efficiency (PCE) of 18.4%, with other device metrics being *V*_oc_ 1.09 V, *J*_sc_ 22.07 mA cm^−2^ and FF of 76.2%. The forward and reverse scan *J*–*V* curves were characterised by a degree of hysteresis that has its origin in interfacial charge accumulation and ion migration processes within the perovskite layer.^29,30^ Interestingly, we observe a monotonic decrease in all *J*–*V* performance parameters (see Fig. S2†) as the amount of KI is increased in the TC perovskite. Indeed, the open-circuit voltage decreased by ∼10 mV for each 5% increase in KI concentration. The *J*_sc_ and FF also underwent a similar reduction with increasing KI concentration, resulting in an almost linear loss of PCE as shown in Fig. S2.† Statistical box plots of all devices are shown in Fig. 1(b) and confirm the trends observed in champion devices that are shown in Fig. 1(a). These results are in contrast to previous reports of improved performance in PSCs following a similar addition of potassium iodide.^23,25^ However, we do observe a reduction in *J*–*V* hysteresis in agreement with previous work.^31,32^ Whilst of limited scientific merit,^33^ the hysteresis index (HI) can be used to quantify this reduction. Here we find that in our best devices, the HI reduces from 0.06 at 0% KI to 0.004 at 20% KI (see Table S3† for further information).

### Characterising film structure and crystallinity

**Fig. 2:**
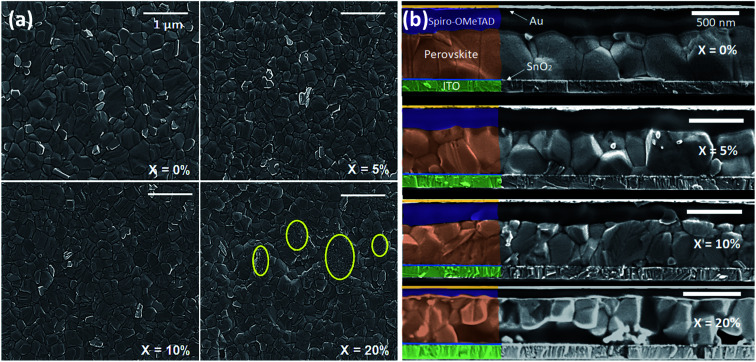
(a) SEM of triple-cation perovskite films containing various fractions of KI (*X* = 0, 5, 10 and 20% KI). Regions marked in yellow denote the presence of a secondary phase in addition to surrounding triple-cation perovskite grains. (b) Cross-section SEM images of TC perovskite devices; *X* = 0, 5, 10 and 20% KI.

We have investigated the effect of KI addition on film microstructure using scanning electron microscopy (SEM) and atomic force microscopy (AFM). An SEM image recorded from a neat TC film is shown in Fig. 2(a). Here, the film is characterised by polycrystalline grains having a size range of 200–600 nm. It is also evident that the film contains regions characterised by excess PbI_2_ that appear as small (∼50–100 nm) crystallites with brighter contrast than the larger (200–600 nm) surrounding perovskite. Fig. 2(a) shows SEM images of perovskite films containing 5, 10 and 20% KI. We find that the addition of 5% KI into the TC perovskite results in a decrease in average grain size from 470 nm (no KI) to 370 nm (5% KI). Interestingly, it appears that the amount of unreacted excess PbI_2_ decreases on addition of KI; a conclusion that is evident from the observed decrease in the fraction of bright PbI_2_ crystallites in Fig. 2(a). As the concentration of KI is increased to 20%, we observe an additional phase (circled yellow in Fig. 2(a)) characterised by a smaller grain structure than is typical for hybrid perovskites. Cross-sectional SEM images of complete devices indicate a similar trend; here ‘neat’ TC films (*X* = 0%) are predominantly composed of columnar grains with vertically aligned grain boundaries as shown in Fig. 2(b). This grain structure is likely to facilitate the transport and extraction of photo generated charge carriers from the device. However, the addition of 5–10% KI in the precursor solution results in the formation of smaller grains than in the control (0% KI) TC device, as shown in Fig. 2(b). Here, the presence of grain boundaries that are oriented parallel to the device substrate may impede vertical charge transport, as well as increasing the bulk trap density through additional grain boundary recombination centres.^34–36^ At the highest KI concentration explored (20%), a secondary phase is evident from the distorted microstructure, as can be seen in Fig. 2(b). We note that our observation of reduced perovskite grain size on addition of KI is in contrast to previous work in which it has been shown that the addition of 5% KI into a perovskite precursor slightly increased film crystallinity and grain size.^23,37,38^ Here, we believe that such differences result from the specific nature of the ETL–perovskite interface. Indeed, when using ETLs that are free from alkali metals, the presence of a small amount of K^+^ ions in the TC solution can lead to an increase in grain size and improved crystallinity due to altered kinetics of crystallization and growth.^39^ However, as the K^+^ ion concentration is increased, a decrease in grain size has also been observed.^39^ This suggests that there likely exists an optimum concentration of K^+^ ions at the ETL–perovskite interface or in the TC solution at which an improvement in grain size can be expected. Indeed, we have observed an increase in grain size in films made on mesoporous TiO_2_ (mp-TiO_2_) ETLs using a TC precursor containing 10% KI (see Fig. S3†). However, the nanoparticle SnO_2_ (np-SnO_2_) colloidal solution used to prepare the ETL already contained a quantity of K^+^ ions in the form of a KOH stabiliser that was added to the solution by the manufacturer.^39,40^ Although the exact concentration of the K^+^ in the np-SnO_2_ ETL solution is unknown, we suspect that the addition of even a small quantity (≥5%) of KI into the TC precursor solution was sufficient to exceed the “sweet spot” concentration for optimum perovskite grain growth and instead causes a reduction in the average perovskite grain size.

We believe that the increased recombination at lateral grain boundaries (in devices containing 5% and 10% KI) together with the presence of secondary non-perovskite phases (in devices containing 20% KI) are significant contributory factors to the observed decrease in the photovoltaic device efficiency.

Analysis of AFM topographs of corresponding perovskite films (see Fig. S4(a–d)†) show increased RMS roughness from 22 nm to 46 nm as the KI concentration is increased from 0% to 20% (see Fig. S5 and Table S4†). Taken together, these observations indicate that KI modifies nucleation-growth dynamics during the formation of TC perovskite films, most likely indirectly through the presence of K^+^ species at the point of crystallisation.

**Fig. 3:**
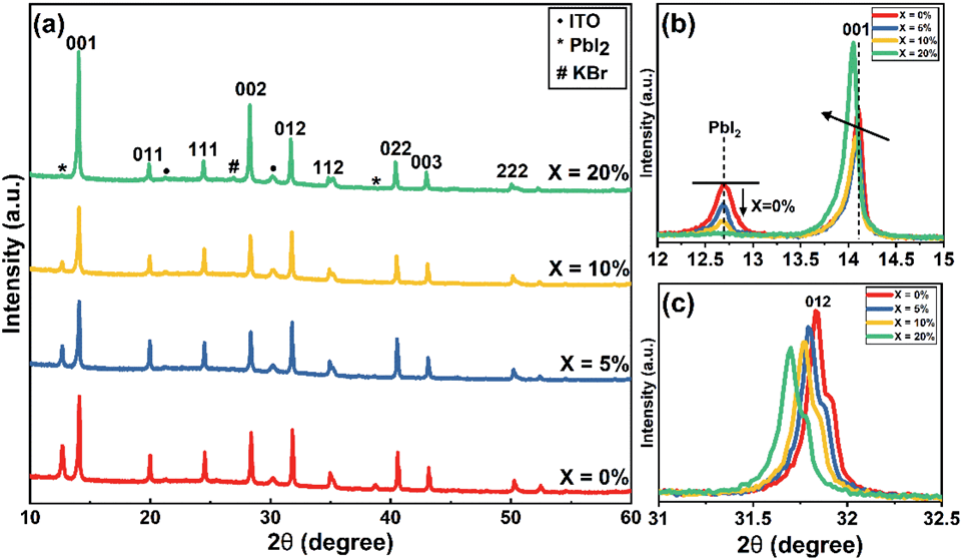
(a) XRD patterns of triple-cation perovskite films with different concentration of KI on ITO/SnO_2_ (ETL) substrates. (b) Magnified view of XRD patterns of TC (*X* = 0–20%) films to show decrease in PbI_2_ peak intensity around 2*θ* = 12.7° and (c) variation in (012) peak around 2*θ* = 31.8° with KI addition.^47^

In order to understand the effect of KI on the crystallisation of the perovskite phase, we used powder X-ray (Cu Kα_avg_ = 1.5419 Å) diffraction (XRD) and grazing-incidence wide-angle X-ray scattering (GIWAXS) to study films containing different concentrations of KI (see Fig. 3 and S6†). In TC composition films without the addition of KI, we observe a diffraction peak at 2*θ* = 12.7° which indicates the presence of excess (unreacted) PbI_2_.^41^ On addition of KI, a systematic decrease in the intensity of this unreacted PbI_2_ peak is observed in Fig. 3(a) (see also Fig. 3(b)); a finding in agreement with previous reports on potassium passivation.^28,42^Fig. 3(c) shows a magnified view of the cubic perovskite (012) scattering peak observed around 2*θ* ∼31.8°. Here, the gradual addition of KI is accompanied by a reduction in the scattering intensity of the (012) peak, together with a shift towards lower 2θ values. This indicates that the addition of KI results in an increase of the unit cell volume, and a reduction of the coherent scattering domain size along the axis of measurement ([001]). Alternately it may point towards a reduction of the vertical grain size;^25^ a conclusion in accord with the grain size analysis performed on the basis of SEM imaging as shown in Fig. 2(a). Note however that the trend of decreased X-ray scattering intensity following the addition of KI is observed in all peaks except for the 20% KI sample, where scattering from the {00*l*} planes show higher intensity. This indicates an altered nucleation and growth due to the addition of KI, resulting in enhanced orientation along the [001] direction. As K^+^ has a smaller ionic size (1.38 Å) compared to the other cations in the TC composition (FA^+^: 2.53 Å, MA^+^: 2.17 Å, and Cs^+^: 1.67 Å), the observed increase in lattice size is consistent with an absence of potassium incorporated at cationic sites.^43,44^

At KI concentrations greater than 5%, we observe scattering features at 2*θ* ∼27.0°, 38.6°, 45.6° and 47.8° (see Fig. S7†) that are consistent with those expected from cubic KBr (ICDD database; card 00-036-1471). This speculation is also confirmed using GIWAXS for 10% and 20% KI samples as shown in Fig. S6(f)† which shows a feature around *Q* ∼1.9 Å^−1^ that corresponds to the (200) reflection of KBr with a *d*-spacing of 3.3 Å. This suggests at high concentrations, the K^+^ from KI preferentially interacts with the Br^−^ species in the TC and results in the formation of KBr. Although we have detected the presence of crystalline KBr, we note that previous reports have assigned this additional phase as a K/Br rich phase (*e.g.* K_2_PbI_4_/KBr_*x*_I_1−*x*_/K_2_PbBr_4_).^25^ We believe that the composition of secondary phases in TC perovskites generated by the addition of KI, are likely to be dependent on subtle differences in processing conditions. Using GIWAXS we also observe the formation of a secondary phase for 10 and 20% KI *via* a peak at ∼0.72 Å^−1^, as shown in Fig. S6(a–d);† a result consistent with previous observations.^25^ We believe that this phase could be a potassium lead halide phase (primarily bromide), and indeed the scattering feature at ∼0.72 Å^−1^ is coincident with features expected from secondary phases induced by the addition of the larger alkali metal rubidium.^41,45^ However, we note that this peak is also coincident with a hydrate phase in TC compositions^46^ observed for devices exposed to moisture during operation, which we discuss later in this paper.

### Optical spectroscopy

We have also studied the UV-vis absorption spectra and steady-state photoluminescence (PL) emission of TC films cast with different KI additive concentrations. Fig. S8(a)† plots PL emission spectra as a function of KI concentration. Here, a gradual redshift in the PL emission peak is observed, going from 755 nm in the neat TC film, to 778 nm at 20% KI. The corresponding thin-film absorption spectra are plotted in Fig. S8(b),† with a similar redshift in band-edge observed with increasing KI. Although strain induced by a change in lattice parameter could lead to change in band-edge energy, we note that Abdi-Jalebi *et al.* did not observe any shift in the absorption or emission wavelength when incorporating KI into pure-iodide TC perovskites, but using scanning transmission microscopy (with energy dispersive X-ray spectroscopy), potassium and bromine rich clusters were formed near the ETL–perovskite interface.^25^ Our diffraction and spectroscopic measurements are therefore consistent with the hypothesis that there is preferential co-ordination between K^+^ and Br^−^ ions in the precursor solution or during crystallisation. This leads to a loss of Br from the perovskite phase as KI is added, thereby depleting the perovskite of Br^−^, with excess iodide from the KI or diminished PbI_2_ phase compensating for the loss of bromide. We note that I^−^ (2.20 Å) has a larger ionic radius than Br^−^ (1.96 Å), therefore a more iodide-rich TC composition is expected to have a larger lattice constant;^43^ a property commensurate with a reduction in the band gap and explaining the redshift in absorption and emission seen here.

**Fig. 4:**
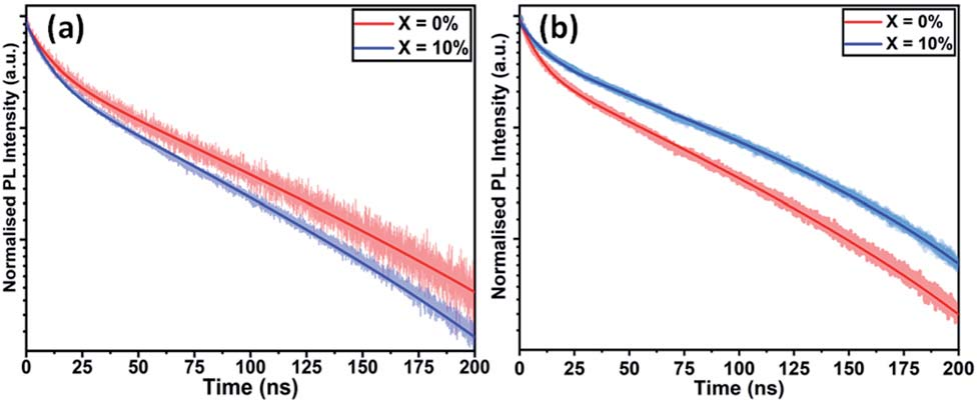
Time-resolved photoluminescence of triple-cation perovskite films with KI added at 0% and 10% cast on (a) ITO/SnO_2_ and (b) FTO/TiO_2_.

To understand the interface photophysics of these perovskites, we have investigated their recombination dynamics when deposited on an ITO/SnO_2_ electron-accepting substrate. In Fig. 4(a), we plot the decay of luminescence following pulsed optical excitation for a series of films to which different concentrations of KI were added to the perovskite precursor. Here, we find a systematic decrease in carrier lifetime as the KI concentration increases, going from 10.8 ns in the neat film, to 8.8 ns in the film prepared from a solution containing an additional 20% KI. This indicates that the presence of potassium does not significantly improve charge carrier extraction when the perovskite is deposited on SnO_2_ (which is efficient in all cases); a result in contrast to previous reports on the beneficial effect of potassium passivation.^23^

### Effect of ETL on PV devices

**Fig. 5:**
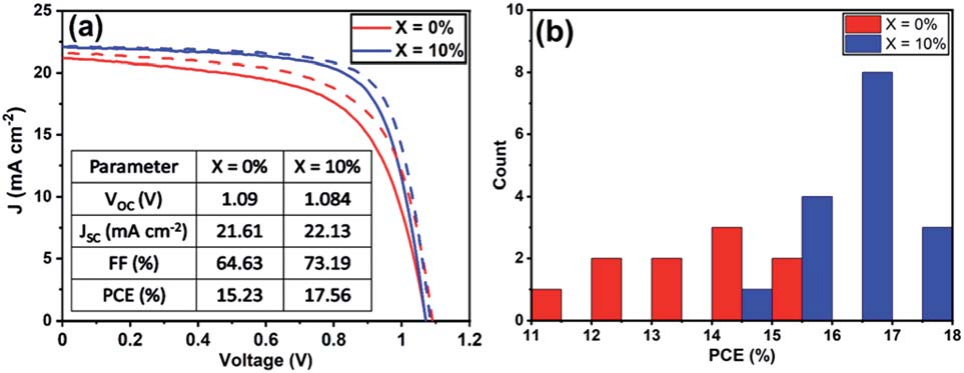
(a) *J*–*V* curves of champion triple-cation perovskite devices using c-TiO_2_/mp-TiO_2_ as the ETL. (b) A histogram of PCE of all TiO_2_ ETL devices from the reverse-scan.

To understand the role played by the substrate in our observations, we have fabricated devices with a TiO_2_ ETL layer, with the perovskite composed of a TC with an addition of 10% KI. This was compared with a control in which no KI was included. Fig. 5(a) plots *J*–*V* curves and performance metrics for devices based on the architecture FTO/compact (c) –TiO_2_/mp-TiO_2_/perovskite/spiro-OMeTAD/Au, with the perovskite containing either 0% or 10% KI. Interestingly, we find that in devices that use a TiO_2_ ETL, there is an improvement in all *J*–*V* metrics with the addition of 10% KI as shown in Fig. S9,† with device PCE increasing from 15.2% to 17.5%. A PCE histogram is shown in Fig. 5(b). X-ray diffraction measurements and UV-vis absorption (shown in Fig. S10 and S11†) demonstrated a similar trend of reduced PbI_2_ scattering intensity and a redshift in absorption following the addition of 10% KI to the perovskite. Importantly, the 10% KI perovskite deposited on TiO_2_ was also characterised by an enhanced carrier lifetime compared to the neat TC case (see Fig. 4(b)); a result in accord with that of Abdi-Jalebi *et al.*^25^ This suggests that the precise nature of the ETL–perovskite interface before passivation plays a significant role in determining the benefit of potassium ion passivation.

To form the SnO_2_ ETL in our n–i–p devices, we used a colloidal SnO_2_ solution in H_2_O that included potassium hydroxide (KOH) as a stabilising agent.^48^ We suspect therefore that the ETL/perovskite interface formed from this material was already ‘moderately’ passivated by the potassium species. Significantly, Bu *et al.* have shown that carrier lifetime can be reduced by removing potassium ions from this SnO_2_ surface; an effect accompanied by impaired device performance.^39^ Interestingly, KOH treatment of a water-washed SnO_2_ layer restored the device performance parameters. Bu *et al.* have also demonstrated that TC perovskite devices based on SnO_2_ layers (processed by chemical bath deposition from a SnCl_2_·2H_2_O solution) could be passivated by ∼3.5 mol% K^+^ in the perovskite solution.^23^ We therefore suggest that, the beneficial effects of KI addition are dependent on the unpassivated interface quality. Here the KI that is added to TC films made on intrinsically passivated SnO_2_ ETLs likely remains in the bulk of perovskite film, forming secondary phases such as KBr. Such interfaces between the perovskite and KBr or other non-perovskite phases may increase the bulk trap density, leading to increased nonradiative recombination, or otherwise hinder charge transfer from the perovskite to the ETL as evidenced in Fig. 4(a). We believe that this scenario explains the observed negative trend in photovoltaic performance parameters on addition of KI to TC perovskite devices made with np-SnO_2._ As mp-TiO_2_ has a high interfacial surface area, it is likely to be characterised by a higher density of trap states than a SnO_2_ ETL. Here, the addition of KI to the TC is expected to passivate traps at the mp-TiO_2_/perovskite interface and thereby improve the performance of devices as observed on addition of 10% KI to the TC. We conclude therefore that the net effect (positive or negative) of the addition of KI to the TC on solar cell performance depends on the extent to which it passivates the ETL–perovskite interface *and/or* generates increased recombination due to the formation of secondary phases within the bulk.

### Stability of devices on addition of KI

Several factors are likely to contribute to the stability of a perovskite solar cell device. These include intrinsic effects (such as the perovskite phase or composition), together with extrinsic factors (such as exposure to moisture, light and oxygen). Other factors that affect device stability include whether (or not) it is run close to its open-circuit voltage or allowed to generate a current by being held at short-circuit. The relative stability of both the ETL and HTL materials used in a device is also important, and this is often dependent on the glass transition temperature of the material. The reactivity and diffusivity of the metal electrode and other dopants or ions can also affect device operational stability. It is therefore important to identify the dominant degradation pathway(s) in a PSC to understand processes that limit its stability. We note that both intrinsic and extrinsic degradation processes can be studied in thin films prepared on representative substrates, while degradation in devices can best be studied *in operando*.

**Fig. 6:**
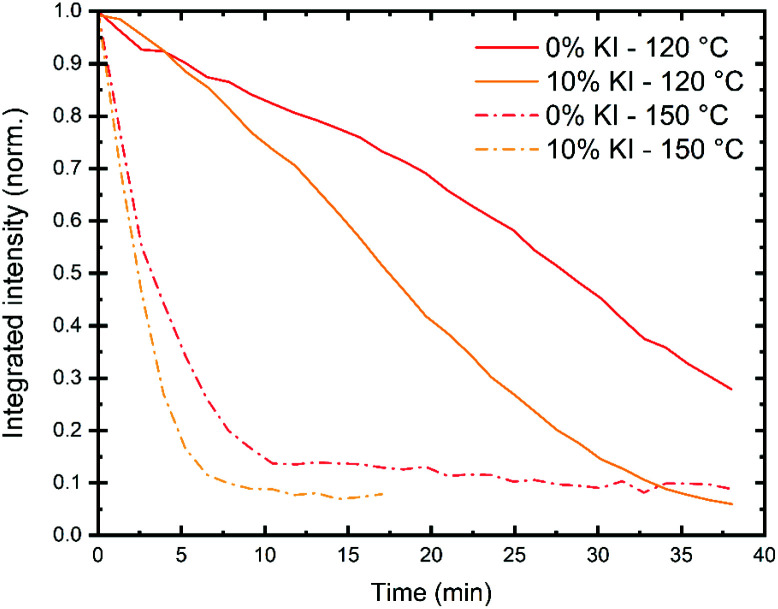
Monitoring the reduction of perovskite crystallinity under accelerated aging conditions using integrated scatter from the (001) reflection for 0% KI (red) and 10% KI (yellow) samples. Films were placed in a sample chamber with a hotplate temperature of either 120 °C (solid lines) or 150 °C (dashed). The measured air temperature (*T*_air_) and relative humidity (RH) for each experiment was *T*_air_ = 59 ± 3 °C, 62 ± 1 °C, 70 ± 2 °C, 69 ± 4 °C and RH = 33 ± 4%, 26 ± 1%, 19 ± 1%, 19 ± 3% for 0% KI, 10% KI at 120 °C and 0% KI, 10% KI at 150 °C, respectively.

To characterise intrinsic material stability, we first performed *in situ* GIWAXS measurements on the triple cation perovskite films deposited on a quartz substrate (0% and 10% KI) to identify intrinsic/extrinsic degradation pathways under accelerated stress conditions. These measurements were performed at the I22 beamline using synchrotron-generated X-rays at the Diamond Light Source. In these experiments, an environmental chamber was integrated into the beamline, allowing us to control temperature, humidity and light-levels. This permitted us to monitor the loss of crystallinity of the perovskite phase during film stressing, with typical 2D X-ray scattering patterns shown in Fig. S12.† Full experimental details are given in the methods section. We first investigated intense damp heat conditions, with high humidity and samples held on a hotplate at either 120 °C or 150 °C, combined with white light illumination controlled to ∼2 suns intensity. In Fig. 6 we show that at 150 °C the integrated scattering intensity from the perovskite (001) reflection decreases rapidly to under 20% after 5–8 minutes for both samples. At 120 °C the loss of intensity is more gradual, with the initial scatter reducing to 50% after 17 min for the 10% KI sample and around 26 min for the 0% KI sample.

**Fig. 7:**
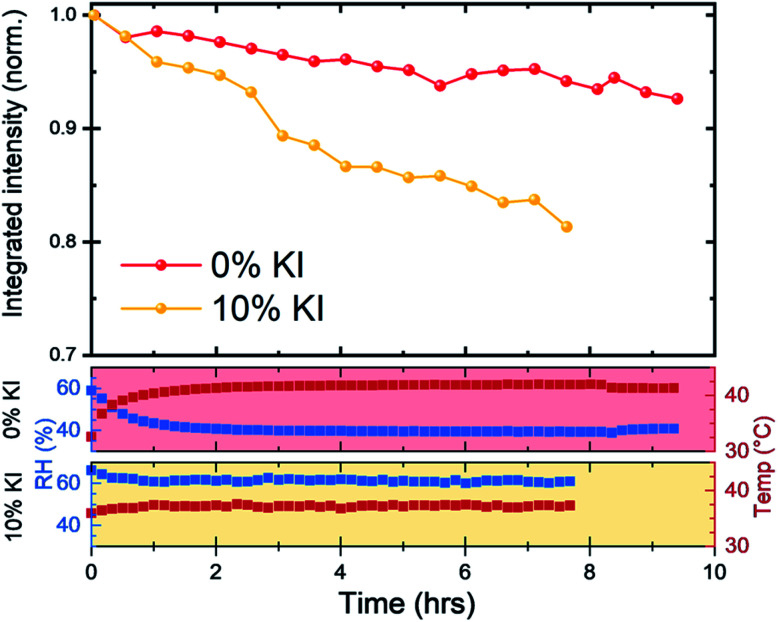
Long term stability of 0% KI and 10% KI films under accelerated aging conditions. Scattering intensity from the (001) reflection was monitored with films kept on a 43 °C hotplate with *T*_air_ and RH monitored. Note, that we recorded small differences in chamber conditions between each measurement run, with average *T*_air_ ≈ 41 °C and RH ≈ 41% for 0% KI and *T*_air_ ≈ 37 °C and RH ≈ 61% for 10% KI as shown in the lower panels, corresponding to absolute humidities of 22 gm^−3^ and 27 gm^−3^, respectively.

We next investigated reduction in scattering intensity of the perovskite phase over an extended period with films on a hotplate set to 43 °C and high humidity conditions (Fig. 7). Again, we observed the same material stability trend, with around 95% of the 0% KI perovskite scattering intensity being retained after 8 hours, whereas the scattering intensity from 10% KI sample had reduced to just under 85% over the same period (although we note small variations in chamber conditions).

Taken together, these observations indicate that the 10% KI film has reduced material stability under accelerated damp heat degradation conditions. We suspect that the origin of such instability results from chemical reactions that occur between the perovskite and water which lead to the formation of hydrates and (in the case of 10% KI) secondary phases such as KBr.^28^ Indeed, Wang *et al.*^49^ have demonstrated that moisture induced degradation in polycrystalline perovskite films initially occurs at grain boundaries, with this degradation then propagating through the film in an in-plane direction.

This suggests that morphological differences in perovskite films (such as reduced grain size) can in fact facilitate moisture induced degradation processes as observed in films produced from 10% KI TC solutions.

**Fig. 8:**
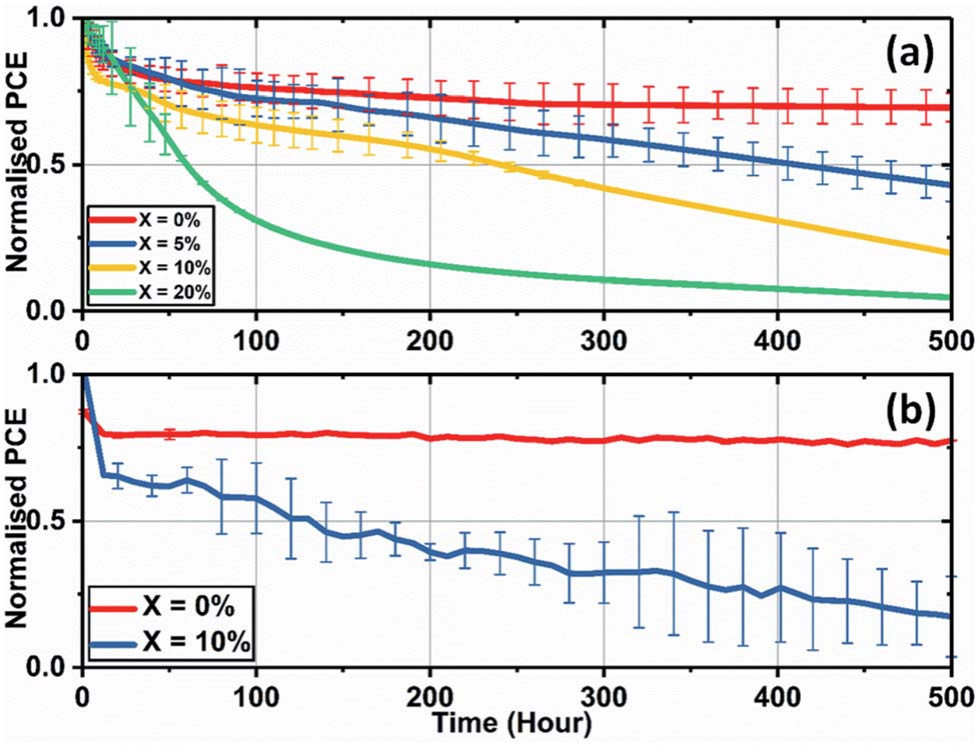
Lifetime stability measurements of triple-cation perovskite devices created from precursor solutions with various levels of KI addition cast on (a) SnO_2_ with data presented from an average value of a minimum of 6 devices, and (b) TiO_2_ having an initial KI concentration of 0 and 10% from an average value of 6 cells.

We have also investigated the effect of adding KI to the perovskite composition on the long-term operational stability of perovskite solar cells. Here, stability measurements were performed in ambient air at a relative humidity of 35–45% and at a temperature of (42 ± 3) °C (induced by the illumination light source). In order to partially limit degradation processes to intrinsic mechanisms, devices were encapsulated using a 100 nm thick layer of SiO_2_ layer to suppress the ingress of moisture and oxygen. During measurement, reverse sweep *J*–*V* curves were recorded approximately every four minutes, with devices being held at *V*_oc_ at other times. Fig. 8(a) shows the temporal evolution of device PCE under continuous illumination for SnO_2_-based PSCs.

Here, the control cells underwent a drop of 25% compared to the initial performance over the first 50 hours of measurement, with the devices then stabilising to 72% of their initial performance after 500 hours of continuous testing. However, devices that included excess KI had reduced stability, with their PCE undergoing a progressive decrease during testing. We have also observed similar trends of reduced operational stability in 10% KI TC perovskite solar cells fabricated using a TiO_2_ ETL (see Fig. 8(b)). This contrasts with control devices (0% KI) on TiO_2_ that exhibit similar stability to TC (0% KI) devices fabricated on SnO_2_.

**Fig. 9:**
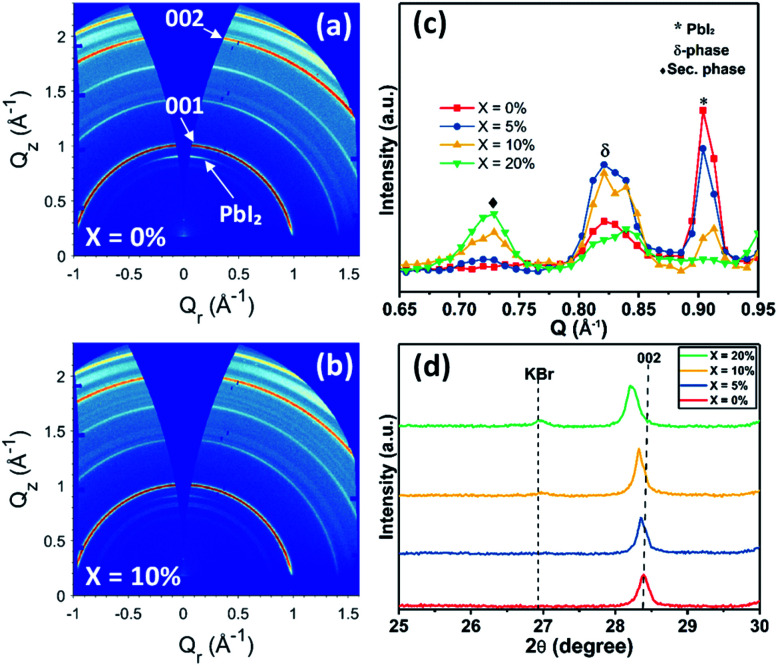
GIWAXS diffraction patterns of aged triple cation perovskite films with (a) 0% KI and (b) 10% KI. (c) Azimuthally integrated profiles from GIWAXS patterns recorded from aged devices (see text for details), highlighting degradation products formed in the region 0.65 ≤ *Q* ≤ 0.95 Å^−1^. (d) XRD patterns from degraded films confirming KBr is still present for 10% and 20% KI addition.

To understand this instability, we prepared devices without SiO_2_ encapsulation and aged them for 50 hours under the same conditions; a result that showed the same trend of reduced stability with increasing KI concentration (see Fig. S13†). We then recorded GIWAXS measurements on the degraded perovskite layers by removing both the Au contact and the spiro-OMeTAD layer. Fig. 9(a) and (b) show the 2D diffraction patterns from TC films having KI added at 0% and 10%, with the same patterns for 5% and 20% KI films shown in Fig. S14† (along with azimuthally integrated diffraction patterns for all four samples). We find that the degraded TC film with 0% KI is characterised by a PbI_2_ peak (∼0.9 Å^−1^), which is oriented in the out-of-plane (*Q*_z_) direction, and is present to a lesser extent at increasing KI concentrations; a result consistent with the undegraded samples (Fig. S6(f)†).

Isotropic scattering rings are also apparent at ∼0.81–0.84 Å^−1^ in both devices, and for all films incorporating KI at ∼0.72 Å^−1^, with Fig. 9(c) highlighting the phases present in the degraded films for each composition. Here, the peak at 0.72 Å^−1^ is ascribed to a secondary or hydrate phase (as observed in the undegraded 10% and 20% KI films) with its intensity being approximately proportional to the additive concentration. The broad feature at ∼0.82 Å^−1^ is attributed to a δ-phase, or more precisely, scattering from the (100) plane of the 2H or 4H hexagonal polytype of the perovskite phase,^50^ which is expected to have peaks in the range 0.81 ≤ *Q* ≤ 0.85 Å^−1^.^45,51^ This is confirmed by two features correlated in intensity with peaks at ∼0.82 Å^−1^ at 1.81 Å^−1^ and with greater intensity at 1.85 Å^−1^, with the latter corresponding well with expected peak positions of either the (202) plane of the 2H polytype or (201) plane of the 4H polytype (see Fig. S15†).^50^ This phase is present to some extent in all films, but is highest in the 5% and 10% KI samples.

Our stability measurements indicate that 20% KI cells undergo critical failure after around 30 hours, whereas the 5% and 10% KI devices declined in efficiency linearly over the testing period (see Fig. S13†). One of the possible origins of the rapid degradation of 20% KI cells could be complete conversion of the perovskite phase to other photo-inactive phases such as δ-FAPbI_3_ (2H polytype) or other secondary phases such as KBr or hydrates. We can in fact rule out this mechanism using the XRD measurements shown in Fig. S16† and UV-vis absorbance shown in Fig. S17.† Here, our measurements indicate the retention of perovskite phase in aged TC devices for all KI compositions. Interestingly, we observe that the device containing a TC with 20% KI undergoes a red-shift in both its absorption and PL emission (780 nm for fresh device *vs.* 800 nm for the aged device (see Fig. S18(b) and (c)†). This process is accompanied by the shift of XRD peaks to lower 2*θ* values, indicating a further increase in the unit cell volume. This suggests that under operational conditions (light illumination and voltage) there is a further loss of bromide (Br^−^) ions from the TC composition, resulting in an additional formation of KBr. This conclusion is further supported by XRD measurements on the aged device where a small increase in intensity of the KBr peak at 2*θ* ∼27° is observed (see Fig. S18(a)† and 9(d)). We note that Zheng *et al.* demonstrated a similar formation of KBr-like compounds under illumination using confocal fluorescence microscopy and STEM-EDX mapping studies on perovskite films containing an addition of KI (3.5%).^52^ Importantly, STEM-EDX elemental mapping results indicated the formation of KBr around the top of the TC film (at the TC/spiro-OMeTAD interface). We suggest this illumination process induces excessive formation of KBr in devices containing a high (20%) KI content. This is likely to lead to increased recombination and may impede charge transport at perovskite–ETL/HTL interfaces, leading to a rapid, critical failure of such devices. It is also apparent that the continual loss in performance of devices containing 5% and 10% KI also suggests that under operational conditions, the perovskite is less phase stable; indeed the formation of KBr and consequent removal of Br from the perovskite phase apparently results in a greater phase instability to a δ-phase.

Our results suggest therefore that while the addition of KI to the perovskite solution has the primary beneficial effect of passivating the ETL/perovskite interface at low concentrations, the addition of KI does not enhance the stability of devices incorporating an np-SnO_2_ electron-extracting contact, due to the fact that the KI is apparently responsible for inadvertent compositional and phase changes to the TC perovskite. We emphasize that our control device (0% KI) that utilised a SnO_2_ ETL was itself moderately passivated at the ETL interface by potassium ions resulting from the KOH stabiliser added to the SnO_2_ deposition solution. This suggests therefore that the presence of small quantities of potassium at the ETL–perovskite interface does not apparently have a negative effect on the stability of a TC PSC device. However, when KI is added in high concentration to a TC precursor solution, it leads to presence of secondary phases in the film. This may cause further changes in the perovskite composition due to the application of a built-in voltage under operational conditions leading to accelerated degradation of TC perovskite solar cells.

## Conclusions

We have systematically explored the effect of adding potassium iodide KI (0–20%) into a triple-cation (TC) perovskite precursor solution to determine whether it can be used to improve the efficiency of solar cell devices by passivating defect states in the perovskite and at the perovskite electron transport layer (ETL) interface. The device ETL was fabricated using a nanoparticle SnO_2_ solution (made using a commercial colloidal product), with a range of KI different concentrations added to the perovskite precursor solution. For comparison, devices containing different concentrations of KI were also fabricated using a TiO_2_ ETL reference. As has been previously observed,^25,52–55^ the addition of KI to the TC perovskite induced a redshift in both PL emission and absorption band-edge, consistent with an increase in lattice parameter. This was accompanied by a reduction in average grain size and increase in film RMS roughness. It was found that devices fabricated on SnO_2_ ETLs incorporating additional KI were characterised by reduced power conversion efficiency compared to un-passivated controls. In contrast, it was found that devices based on a TC perovskite incorporating 10% KI and fabricated on a TiO_2_ ETL had a relatively improved power conversion efficiency compared to a 0% KI control. We assign the lack of improvement in the performance of SnO_2_ based solar cells with a KI additive to the fact that the SnO_2_/perovskite interface was already partially passivated by the KOH stabiliser that was present in the colloidal dispersion. Indeed, we suspect that this stabiliser acts as an intrinsic source of K^+^ ions that minimises defects at the ETL/perovskite interface. For this reason, the addition of excess KI to the TC precursor solution resulted in a reduction in device performance due to the segregation of KBr and the formation of other non-perovskite phases.

We investigated the effect of this KI additive on both intrinsic film stability and on device performance over 500 hours of illumination and bias and found it has a detrimental effect on operational stability in devices incorporating both SnO_2_ and TiO_2_ ETLs. At a KI concentration of 5 and 10% KI in the initial precursor, the perovskite is found to be less phase stable, with the formation of KBr and consequent removal of Br from the perovskite phase during film formation leading to a greater phase instability to a δ-phase under operational conditions. At very high KI concentrations (20% or higher) the perovskite devices undergo rapid, critical failure due to additional extraction of bromide species and the formation of a non-perovskite phase (KBr) under illumination and applied bias.

## Conflicts of interest

D. G. L. is a co-director of the company Ossila that retail materials and equipment used in perovskite photovoltaic device research and development.

## Supplementary Material

RA-010-D0RA07107B-s001
