# Supplementary material for: Potassium iodide reduces the stability of triple-cation perovskite solar cells
Source: RSC Adv. 2020 Nov 6;10(66):40341–50. doi: 10.1039/d0ra07107b (PMC9057489; doi:10.1039/d0ra07107b)
Supplement: RA-010-D0RA07107B-s001 [file RA-010-D0RA07107B-s001.pdf]

Electronic Supplementary Material (ESI) for RSC Advances.
This journal is © The Royal Society of Chemistry 2020

Electronic Supporting Information (ESI)

Potassium Iodide Reduces the Stability of Triple-Cation Perovskite Solar Cells

Tarek I. Alanazi[a,b], Onkar S. Game[a], Joel A. Smith[a], Rachel C. Kilbride[a], Claire Greenland[a], Rahul Jayaprakash[a], Kyriacos Georgiou[a], Nicholas J. Terrill[c] and David G. Lidzey[a*]

[a.] Department of Physics and Astronomy, University of Sheffield, Sheffield, S3 7RH, U.K.

[b.] Department of Physics, College of Science, Northern Border University, Arar, 73222, KSA

[c.] Diamond Light Source, Harwell Science and Innovation Campus, Fermi Ave, Didcot, Oxfordshire OX11 0DE, U.K.

[*] Corresponding author: d.g.lidzey@sheffield.ac.uk

Methods

Device fabrication

Materials and cleaning process. The following materials were purchased from Ossila Ltd: Indium tin oxide (ITO) glass substrates (20 Ω/square), FTO (6 Ω/square), formamidinium iodide (FAI, 98%), spiro-OMeTAD (sublimed 99.5%) and methylammonium bromide (MABr, 99.5%). Additional materials were purchased as follows: SnO_2 15 wt% in H_2O colloidal dispersion liquid (Alfa Aesar 44592.36), lead (II) bromide $PbBr_2$, lead (II) iodide (PbI_2, 99.99%) (Tokyo Chemical Industry UK Ltd) and gold wire (Cookson Gold Ltd). All other solvents and materials were purchased from Sigma Aldrich Ltd.

ITO and FTO were etched using a 4M of hydrochloric acid solution together with zinc powder that was scattered onto the substrate surface. Following etching, substrates were immersed in a solution of 2% Hellmanex mixed with boiling deionised water and then sonicated for 15 minutes, before rinsing with DI water. This was followed by sonication in isopropanol for a further 15 minutes. Finally, substrates were dried using a nitrogen jet and cleaned for 15 minutes using an ultraviolet-ozone (UV) treatment.

Electron transport layer. Both SnO_2 and TiO_2 were used as electron transport layers (ETLs). SnO_2 was deposited onto ITO by spin-coating at 3000 rpm from a commercial (SnO_2 colloidal solution 15 wt% in water) which was diluted with DI water (6.5:1). Following deposition, the film was annealed for 30 minutes at 150°C. Compact TiO_2 (c-TiO_2) was deposited on top of FTO substrates by spray pyrolysis. Here, the FTO was heated to 450 °C with titanium diisopropoxide bis(acetylacetonate) (75 wt% in isopropanol) dissolved in n-butanol (1:9) sprayed onto its surface, followed by a further 30 minutes of annealing before cooling to room temperature. Following this, mesoporous TiO_2 (mp-TiO_2) was deposited on-top of the c-TiO_2 by spin coating TiO_2 paste dissolved in n-butanol (1:6) at 5000 rpm for 30 seconds, following which it was annealed at 450 °C for 45 minutes.

Perovskite. Perovskite triple-cation (TC) precursor solution was prepared by dissolving FAI (1M), MABr (0.2M), $PbBr_2$ (0.2M) and PbI_2 (1.1M) in anhydrous DMF/DMSO (4:1 volume ratio), into which an additional 42 µl (per ml of solvents) of 1.5M CsI solution in DMSO was added. Following this, 1.5M potassium iodide (KI) solution in DMF/DMSO (4:1 volume ratio)

was added at various percentages (0-20%). The TC precursor solution was then deposited on top of the ETL. Here, films were spin coated using a two-step process at 1000 rpm for a duration of 10 seconds, followed by 6000 rpm for 20 seconds, with 100 µl of chlorobenzene antisolvent dropped onto the films after a duration of 25 seconds. Finally, the films were annealed at 100°C for 60 minutes.

Hole transport layer and top contact. The hole transport layer (HTL) was prepared from a solution containing 86 mg ml^{-1} spiro-OMeTAD in chlorobenzene, doped with 34 µl of 4-tert-butylpyridine (tBP), 20 µl of bis(trifluoromethane)sulfonimide lithium (LiTFSI) in acetonitrile solution (500 mg/mL) and 11 µL of FK209 Co(II) PF6 (FK209) in acetonitrile solution (300 mg/mL). After mixing and filtering, the HTL solution was spin-coated dynamically at 4000 rpm for 30 seconds before leaving in dry air overnight. The device cathode (Au) was thermally evaporated under high vacuum creating a 80 nm thick layer. Where used, SiO_2 encapsulation was deposited by electron-beam evaporation under high vacuum at a deposition rate of 0.1-1 Å s^{-1} to a final thickness of 100 nm. The structure of a typical device is shown schematically in Figure S1.

Characterisation

Current-voltage measurement. To characterise device performance, JV characteristics were recorded when illuminated using a Newport 92251A-1000 AM1.5g solar simulator. The system was initially calibrated using an NREL certified silicon reference cell, with the simulator optical power output adjusted to 100 $mWcm^{-2}$ at 25°C. During measurement, an aperture mask (0.026 cm^2) was placed over each individual solar cell to accurately define the areas over which the perovskite solar cell was illuminated and to reduce contributions resulting from stray (scattered) light. JV curves were recorded using a Keithley 237 source measure unit that swept the voltage (in both directions) from -0.1 to 1.2 V at 0.1 Vs^{-1}.

Scanning electron microscopy (SEM). SEM images of perovskite surfaces and device cross-sections were recorded using a Carl Zeiss-modified Raith Nanofabrication SEM with an in-lens detector. Here the microscope was operated with a typical working distance of ~2.5 mm and at a low accelerating voltage of 1.5 kV to minimise sample damage. Grain sizes were calculated using ImageJ software.

Atomic Force Microscopy (AFM). Images were recorded using a Veeco Dimension 3100 instrument with a Nanoscope 3A feedback controller used in tapping mode. The AFM was equipped with TESPA-V2 probes having a resonance frequency of around 320 KHz and spring constant of 42 Nm^{-1}.

X-ray diffractometer. A PANalytical X'Pert Pro system powered by a Philips PW3050/60 (θ/ θ) X-ray generator diffractometer (240 mm diameter) with a PW3064 sample spinner was used to determine X-ray diffraction data. The X-ray source was a Copper Line Focus X-ray tube with Kα radiation ($K\alpha_1$ = 1.5406 Å, $K\alpha_2$ = 1.5444 Å, Kα ratio 0.5, $K\alpha_{avg}$ = 1.5419 Å) and a Ni Kβ absorber (0.02 mm, Kβ = 1.3923 Å). This was run at 45 kV with a tube current of 40 mA. For data collection, a 1D-detector was used in Bragg-Brentano geometry, with data collected from 5.00° to 80.00° 2θ with a step size of 0.0131°. All scans were carried out continuously in intervals of 0.31 seconds.

Photoluminescence. A 405 nm CW laser diode was used to excite the samples. Photoluminescence was collected at a normal incidence using an optical fibre and was directed into an Andor Shamrock CCD spectrometer.

Time-Resolved photoluminescence (TRPL). Time-correlated single-photon counting (TCSPC) was used to measure the transient luminescence emitted from the perovskite films. Here, the samples were excited with a 507 nm pulsed laser (PicoQuant GmbH) at 2.5 MHz with a pulse width of ~600 ps and an excitation fluence of ~1 $\mu J\ cm^{-2}$. Luminescence was detected using a Micro Photon Devices silicon SPAD (single photon avalanche diode). The system was controlled using a TimeHarp 260 Pico PCIe board (PicoQuant GmbH) with data points having a time resolution of 25 ps.

Grazing incidince wide-angle X-ray scattering (GIWAXS). Xeuss 2.0 (Xenocs) system with 9.243 keV X-rays from a liquid Ga MetalJet source (Excillum) was used to perform ex-situ grazing incidence X-ray scattering. X-rays were incident on the perovskite films at a grazing angle of 2°. The sample and flight tube were held under vacuum during operation to remove

background scatter. A Pilatus3R 1M detector held at a distance of ~300 mm from the sample was used to detect scattered X-rays. The GIXSGUI MATLAB toolbox was used for data analysis, reduction and reshaping.[1]

In-situ GIWAXS experiments were performed at the I22 beamline at Diamond Light Source. Here, X-rays with photon energy 14 keV were incident on thin film samples having the structure glass/ITO/SnO_2/perovskite which were held inside a sample chamber. This controlled environmental chamber contained a resistive heater in the base which was set to 43, 120 or 150 °C for each experiment. This was combined with illumination from a white light LED set to ~2 suns intensity (200 ± 10 mW/cm^2), with humidity controlled by using a moisture bubbler on a hotplate, fed from a dry N_2 feed, as used in our previous work.[2] Scattering was collected with a PILATUS P3-2M-L (DECTRIS) hybrid photon counting detector mounted approximately 260 mm from the sample, with all integrated intensities averaged from two locations on each sample. Beam damage was carefully controlled (attenuation and maximum exposure) under accelerated aging conditions prior to each experiment. This allowed us to ensure that material changes observed were caused by the designed degradation factors rather than from X-ray beam damage. Data was reduced and analysed using *PyFAI*.[3]

Lifetime testing. An Atlas Suntest CPS+ with a 1500 W xenon bulb equipped with quartz IR reducing filters calibrated to 100 mWcm^{-2} was used to continuously irradiate samples in ambient air (typically ~35 - 45% RH). Devices were irradiated without an aperture mask in place. *J-V* measurements were continually recorded under reverse sweep from 1.2 to 0 V at a scan rate of 0.012 V s^{-1}. Devices were held at open circuit between measurements, with scans on each cell recorded at intervals of approximately 4 minutes. Aged perovskite devices used for GIWAXS, XRD and UV-Vis absorption measurements (data shown in Figures 7(a), 7(b), S12, S14, S15 and S16) were aged using the same lamp system and under the same conditions, however devices were not protected using a SiO_2 encapsulation layer.

Supplementary Figures a

Table S1 Comparison of th
halide perovskite.

ETL	Perovskite composition	Potassium content (mol%)	Improved parameters (H = reduced hysteresis)					Aging protocol	Device stability condition	Stability improvement (Yes/No)	Ref
			V_{OC}	J_{SC}	FF	PCE	H				
c-TiO_2 | mp-TiO_2	$FA_{0.85}MA_{0.10}Cs_{0.05}PbI_{2.7}Br_{0.3}$	~ 1%	✓	✓			✓	N/A			**3**
c-TiO_2 l mp-TiO_2	$(Cs,FA,MA)Pb(I_{0.85}Br_{0.15})_3$	10%	✓	✓	✓	✓	✓	**Shelf-life** (ISOS D-1)	40 °C, N_2 atmosphere	Yes	**4**
c-TiO_2 l mp-TiO_2	$(Cs,FA,MA)Pb(I_{0.85}Br_{0.15})_3$	10%	✓	✓	✓	✓	✓	**Operational** MPP (ISOS L-1)	40 °C, 1 sun, N_2 atmosphere	N/A	**4**
c-TiO_2 l mp-TiO_2	$(Cs_{0.06}MA_{0.15}FA_{0.79})Pb(I_{0.85}Br_{0.15})_3$	10%	✓	✓	✓	✓	✓	N/A			**5**
SnO_2 (CBD)	$Cs_{0.05}(FA_{0.85}MA_{0.15})_{0.95}Pb(I_{0.85}Br_{0.15})_3$	3.5%	✓	✓	✓	✓	✓	N/A			**6**
TiO_2	$Cs_{0.925}K_{0.075}PbI_2Br$	0.075%		✓	✓	✓		**Shelf-life** (ISOS D-1)	RH=20%, 20 °C	Yes	**7**
c-TiO_2:LiMg l mp-TiO_2:Li	$FA_{0.85}MA_{0.15}Pb(I_{0.85}Br_{0.15})_3$	5%	✓	✓	✓	✓	✓	**Shelf-life** (ISOS D-1)	Dark drying room, dew point temp. -30 °C	N/A	**8**
PCBM (pin)	$MAPbI_3$ (PEDOT:PSS HTL)	0.5%	✓		✓	✓	✓				**9**
TiO_2	$MAPbI_3$	N/A	✓		✓	✓					**10**
TiO_2	$FA_{0.87}MA_{0.13}Pb(I_{0.87}Br_{0.13})_3$	5%	✓	✓	✓	✓	✓	**Shelf-life** (ISOS D-1+light)	RH=30%, room temp., 1 sun	Yes	**11**
np-SnO_2 (Alfa Aesar)	$Cs_{0.05}FA_{0.79}MA_{0.16}Pb(I_{0.75}Br_{0.25})_3$	15%	✓	✓	✓	✓	✓	**Shelf-life** (ISOS D-1)	RH=20%, ambient air	yes	**12**
0.1 M solution of $SnCl_2$ (98%) in absolute ethanol (SnO_2)	$MA_{0.17}FA_{0.83}PbI_{2.5}Br_{0.5}$	3%	✓	✓	✓	✓	✓	**Shelf-life** (ISOS D-1)	RH=30-45%, ambient air	Yes	**13**
SnO_2 (CBD)	$Cs_{0.05}(FA_{0.85}MA_{0.15})_{0.95}Pb(I_{0.85}Br_{0.15})_3$	3.5%	✓	✓	✓	✓	✓	**Shelf-life** (ISOS D-1)	RH=10±5%, ambient air	Yes	**14**
c-TiO_2 l mp-TiO_2	$MAPbI_3$	0.5%	✓	✓	✓	✓	✓	**Shelf-life** (ISOS D-1)	RH=46%, ambient air	Yes	**15**
SnO_2 l c-TiO_2 l mp-TiO_2	$(FA_{0.83}MA_{0.17})Pb(I_{0.83}Br_{0.17})_3$	3%	✓	✓	✓	✓	✓	**Shelf-life** (ISOS D-1)	RH=40%, 25 °C	Yes	**16**
np- SnO_2 (Alfa Aesar)	$Cs_{0.05}FA_{0.79}MA_{0.15}PbI_{2.45}Br_{0.55}$	10%					✓	**Operational** (ISOS L-1 and held at V_{oc})	**RH~40% @ 42 °C, 1 sun**	**No**	**This work**
c-TiO_2 | m-TiO_2	$Cs_{0.05}FA_{0.79}MA_{0.15}PbI_{2.45}Br_{0.55}$	10%		✓	✓	✓	✓	**Operational** (ISOS L-1 and held at V_{oc})	**RH~40% @ 42 °C, 1 sun**	**No**	**This work**

References for Table 1

1 D.-Y. Son, S.-G. Kim, J.-Y. Seo, S.-H. Lee, H. Shin, D. Lee and N. Park, *J. Am. Chem. Soc.*, 2018, **140**, 1358–1364.

2 M. Abdi-Jalebi, Z. Andaji-Garmaroudi, S. Cacovich, C. Stavrakas, B. Philippe, J. M. Richter, M. Alsari, E. P. Booker, E. M. Hutter, A. J. Pearson, S. Lilliu, T. J. Savenije, H. Rensmo, G. Divitini, C. Ducati, R. H. Friend and S. D. Stranks, *Nature*, 2018, **555**, 497–501.

3 M. Abdi-Jalebi, Z. Andaji-Garmaroudi, A. J. Pearson, G. Divitini, S. Cacovich, B. Philippe, H. Rensmo, C. Ducati, R. H. Friend and S. D. Stranks, *ACS Energy Lett.*, 2018, **3**, 2671–2678.

4 F. Zheng, W. Chen, T. Bu, K. P. Ghiggino, F. Huang, Y. Cheng, P. Tapping, T. W. Kee, B. Jia and X. Wen, *Adv. Energy Mater.*, 2019, **9**, 1901016.

5 J. K. Nam, S. U. Chai, W. Cha, Y. J. Choi, W. Kim, M. S. Jung, J. Kwon, D. Kim and J. H. Park, *Nano Lett.*, 2017, **17**, 2028–2033.

6 Z. Tang, T. Bessho, F. Awai, T. Kinoshita, M. M. Maitani, R. Jono, T. N. Murakami, H. Wang, T. Kubo, S. Uchida and H. Segawa, *Sci. Rep.*, 2017, **7**, 12183.

7 J. Chang, Z. Lin, H. Zhu, F. H. Isikgor, Q.-H. Xu, C. Zhang, Y. Hao and J. Ouyang, *J. Mater. Chem. A*, 2016, **4**, 16546–16552.

8 W. Zhao, Z. Yao, F. Yu, D. Yang and S. F. Liu, *Adv. Sci.*, 2018, **5**, 1700131.

9 L. Kuai, Y. Wang, Z. Zhang, Y. Yang, Y. Qin, T. Wu, Y. Li, Y. Li, T. Song, X. Gao, L. Wang and B. Sun, *Sol. RRL*, 2019, **3**, 1900053.

10 L. Wang, G. Wang, Z. Yan, J. Qiu, C. Jia, W. Zhang, C. Zhen, C. Xu, K. Tai, X. Jiang and S. Yang, *Sol. RRL*, 2020, **4**, 2000098.

11 D. Yao, C. Zhang, N. D. Pham, Y. Zhang, V. T. Tiong, A. Du, Q. Shen, G. J. Wilson and H. Wang, *J. Phys. Chem. Lett.*, 2018, **9**, 2113–2120.

12 T. Bu, X. Liu, Y. Zhou, J. Yi, X. Huang, L. Luo, J. Xiao, Z. Ku, Y. Peng, F. Huang, Y.-B. Cheng and J. Zhong, *Energy Environ. Sci.*, 2017, **10**, 2509–2515.

13 S. Jia, J. Wang and L. Zhu, *J. Mater. Sci. Mater. Electron.*, 2019, **30**, 2057–2066.

14 Y. Yang, L. Wu, X. Hao, Z. Tang, H. Lai, J. Zhang, W. Wang and L. Feng, *RSC Adv.*, 2019, **9**, 28561–28568.

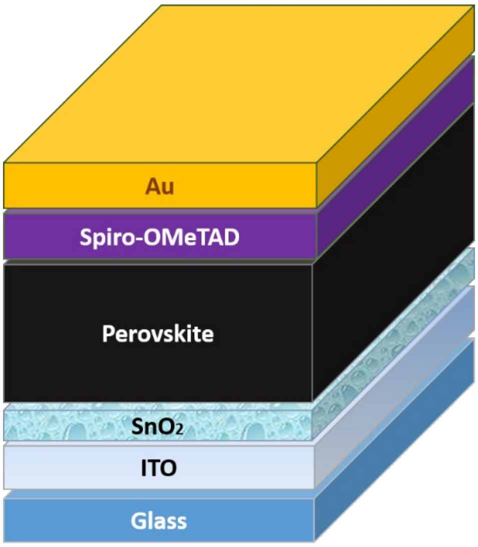

Fig S1 Standard architecture (*n-i-p*) triple-cation PSCs having the structure ITO/SnO_2/perovskite/spiro-OMeTAD/Au.

Concentration of KI	V_{OC} (V)	J_{SC} (mA cm^{-2})	FF (%)	PCE(%)
X = 0%	1.10 (1.08 ± 0.01)	22.83 (22.33 ± 0.41)	77.62 (75.68 ± 1.32)	18.38 (17.51 ± 0.48)
X = 5%	1.08 (1.07 ± 0.01)	22.55 (21.27 ± 0.18)	74.78 (73.55 ± 0.95)	17.36 (16.39 ± 0.61)
X = 10%	1.07 (1.05 ± 0.01)	21.47 (21.21 ± 0.16)	74.53 (72.49 ± 1.38)	16.85 (15.90 ± 0.74)
X = 20%	1.06 (1.03 ± 0.03)	20.48 (20.18 ± 0.24)	72.53 (71.18 ± 1.02)	15.53 (14.72 ± 0.72)

Table S2 Summary of reverse scan (V_{OC} to J_{SC}) performance metrics of all devices and average values from a minimum of 17 cells per testing condition.

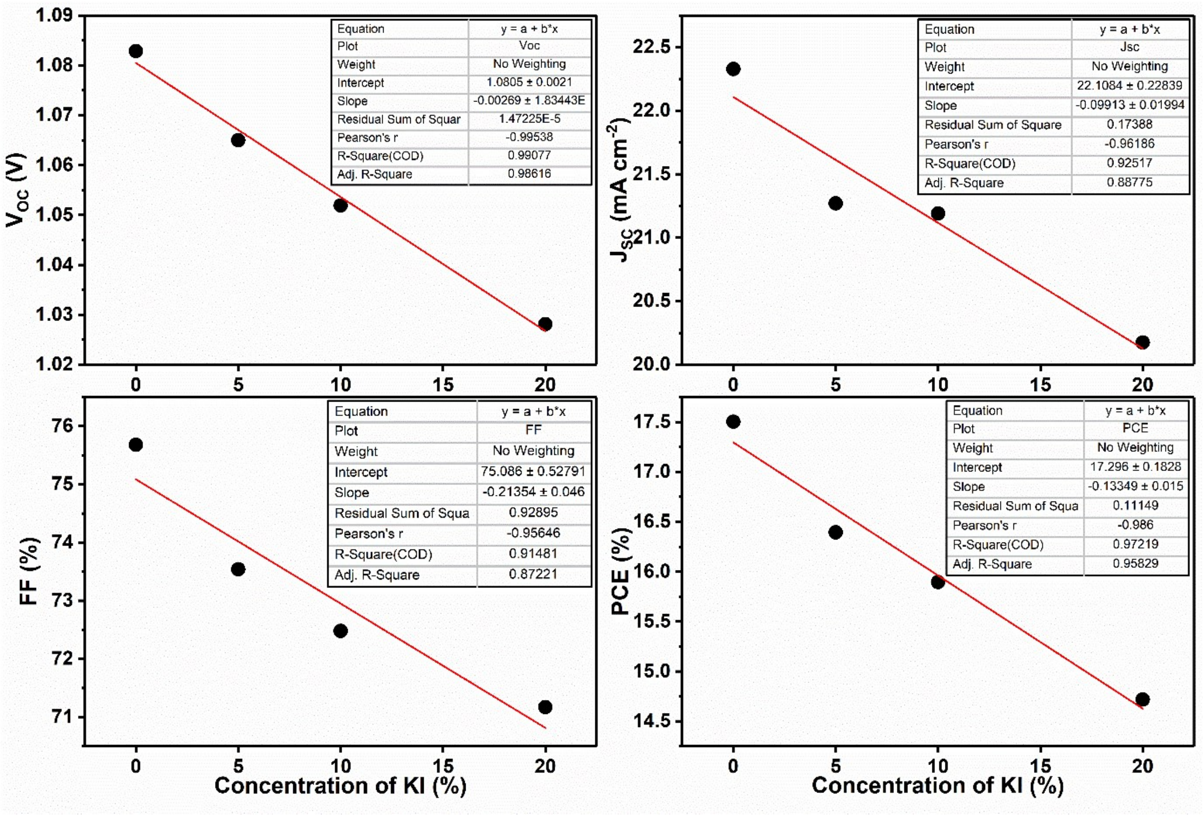

Fig S2 Trend in the mean performance metrics of devices as a function of KI concentration.

KI addition	Hysteresis Index
X = 0%	0.0598
X = 5%	0.0138
X = 10%	0.0065
X = 20%	0.0039

Table S3 *J-V* hysteresis index (HI) for champion devices whose metric are presented in Table S1 (main manuscript). HI is calculated using $HI = \frac{PCE\ (reverse) - PCE\ (forward)}{PCE\ (reverse)}$ [4].

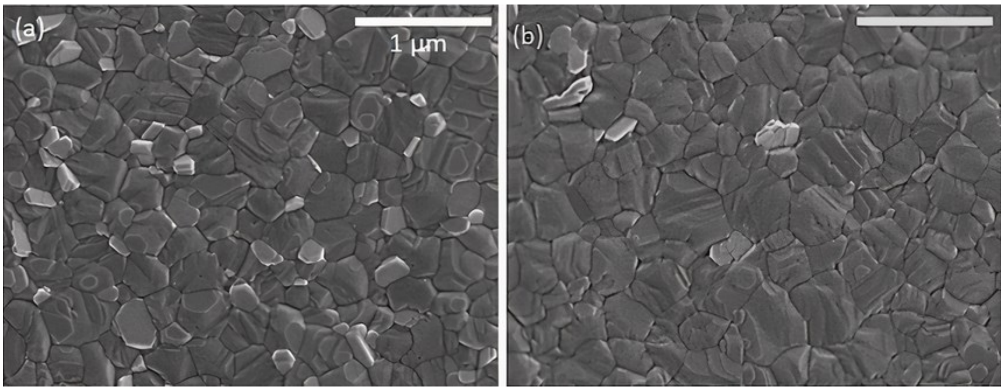

Fig S3 SEM images of triple-cation perovskite films on mp-TiO_2; (a) pristine film and (b) X = 10% KI.

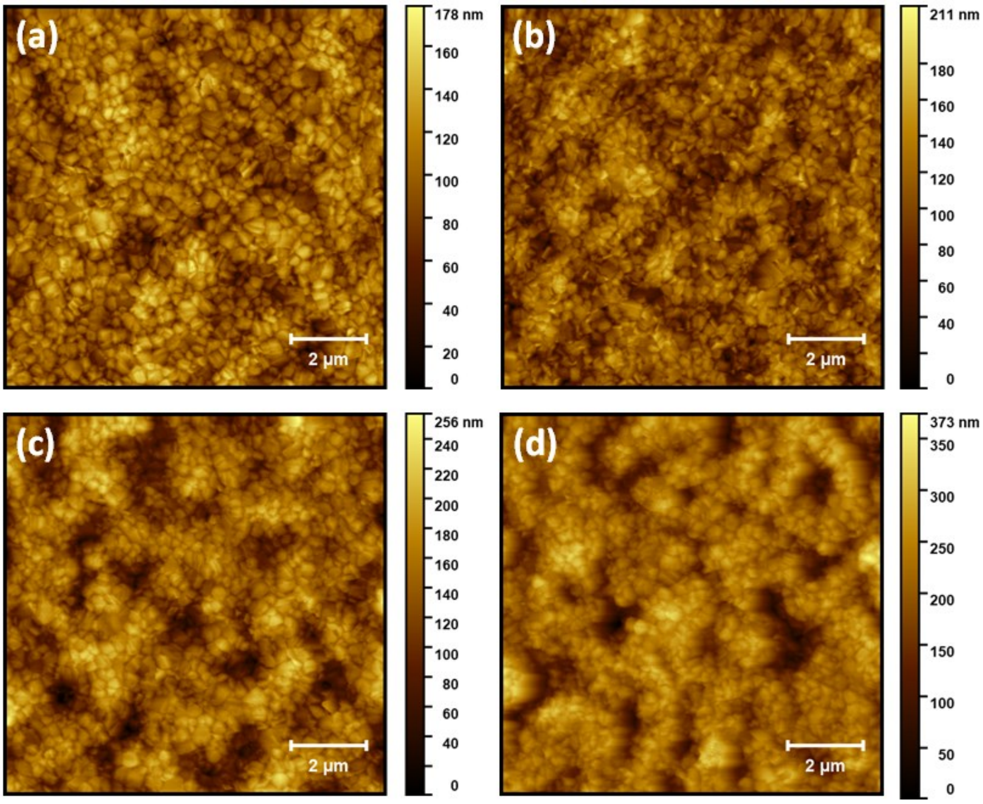

Fig S4 AFM images of triple-cation perovskite films; (a) pristine film, and with KI added at different concentrations (b) X = 5%, (c) X = 10%, and (d) X = 20% KI.

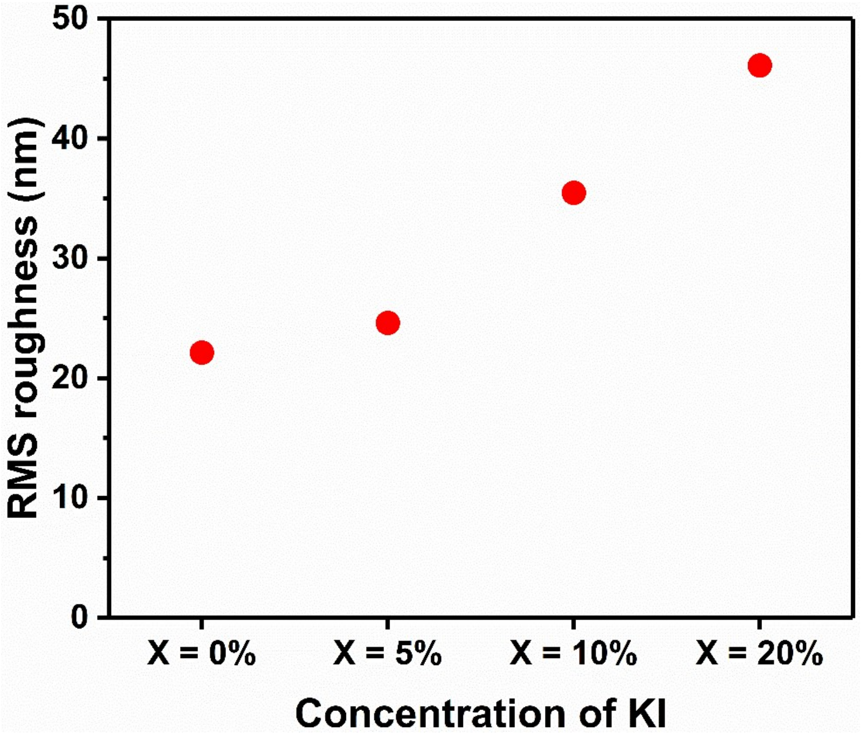

Fig S5 Root-mean-square roughness of films increases as KI concentration increases.

Composition	RMS roughness (S_q) (nm)	Mean roughness (S_a) (nm)
X = 0%	22.13	17.78
X = 5%	24.61	19.81
X = 10%	35.47	28.65
X = 20%	46.09	36.31

Table S4 Root-mean-square roughness RMS (S_q) and mean roughness (S_a) as determined from AFM images.

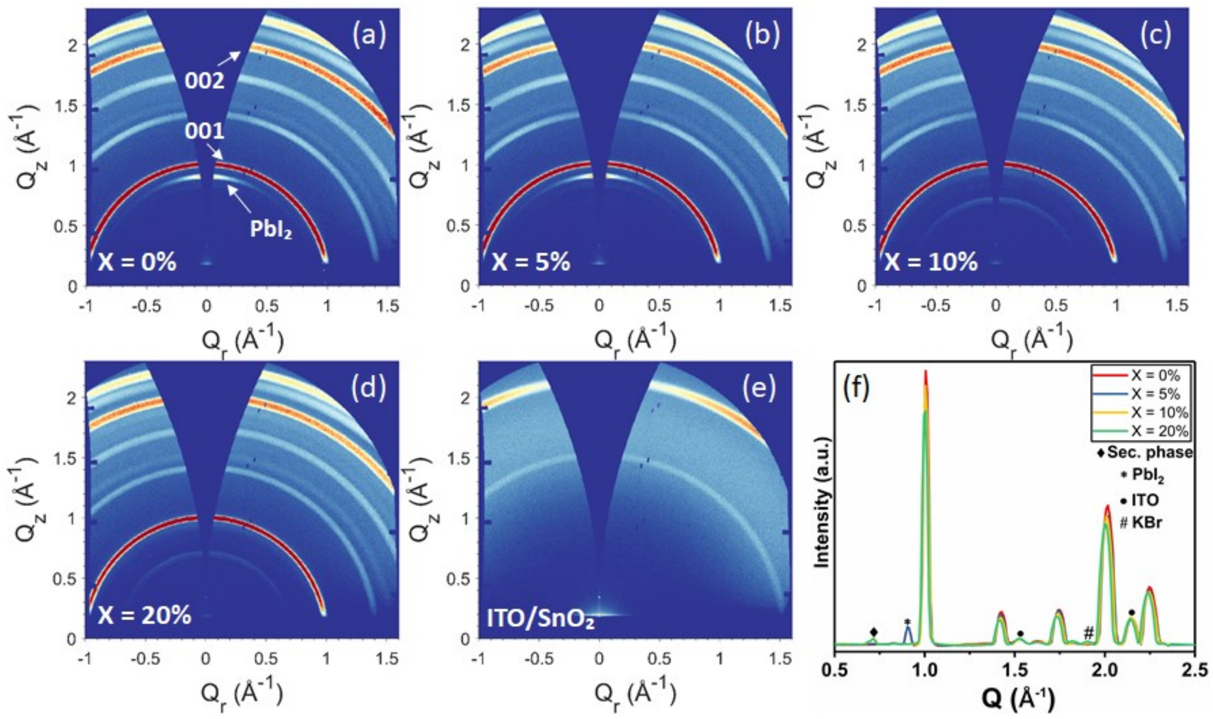

Fig S6 (a)-(d) GIWAXS measurements of fresh triple-cation perovskite films collected at 2° grazing incidence angle with different concentration of KI, (e) the diffraction pattern of (amorphous) SnO_2 on top of (crystalline) ITO and (f) azimuthal integrations of GIWAXS patterns for different concentration of KI.

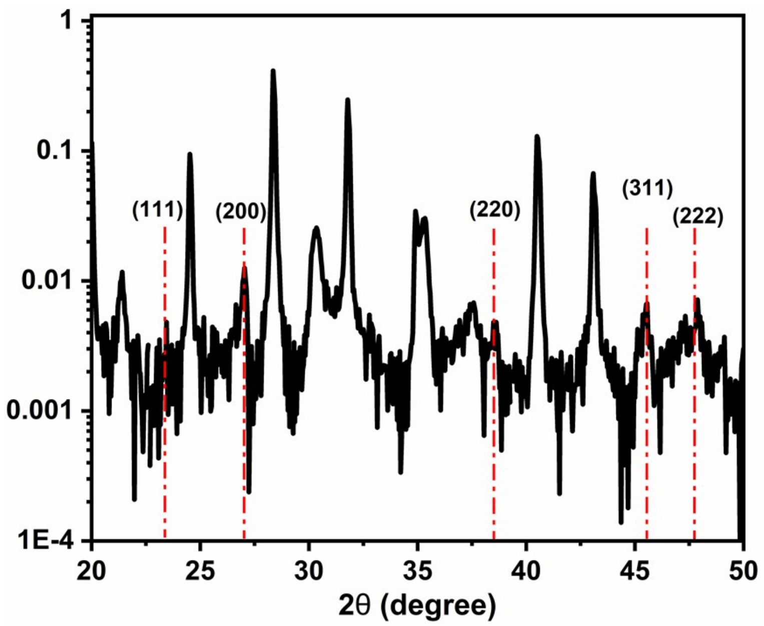

Fig S7 Slow XRD scan of 20% KI plotted on log-linear scale. Red vertical lines show the standard position of KBr peaks (ICDD database; card 00-036-1471).

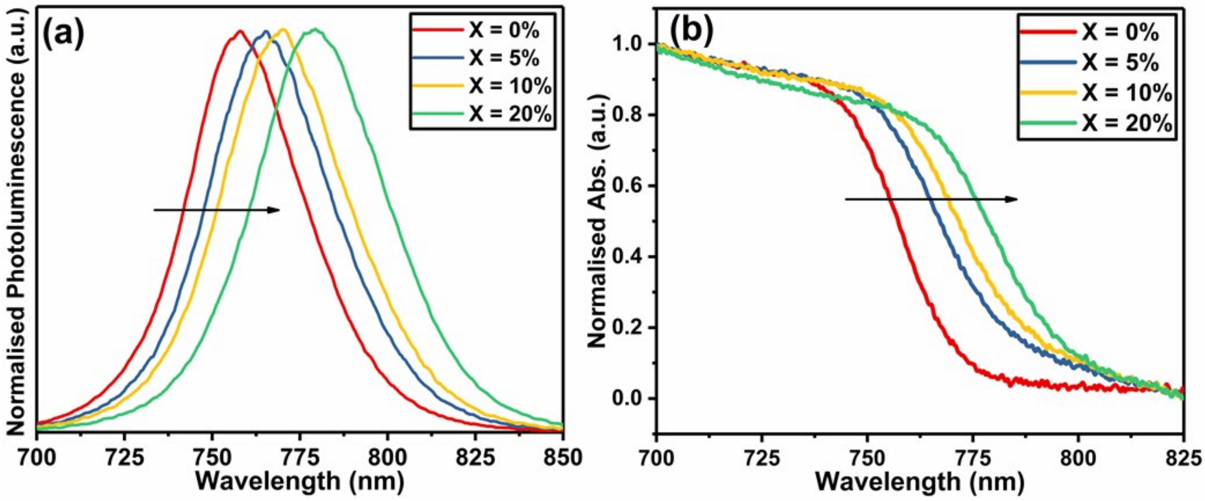

Fig S8 Normalised (a) photoluminescence and (b) UV-Vis absorption for triple-cation perovskite films with X = 0, 5, 10 and 20% KI added.

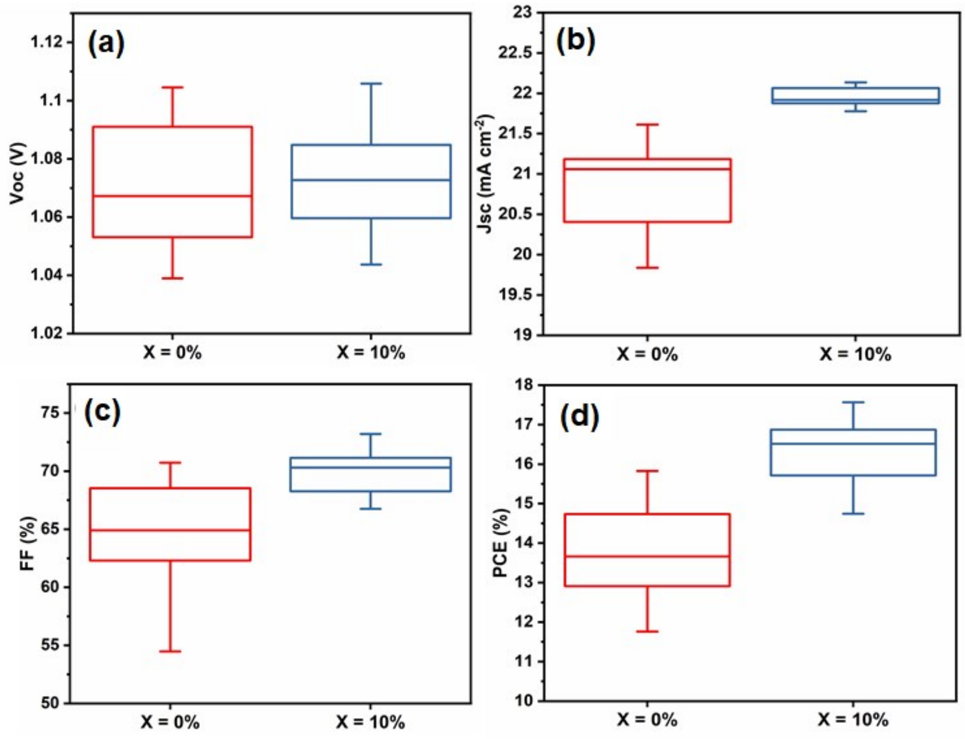

Fig S9 Box plots detailing the performance of all TiO_2 ETL devices.

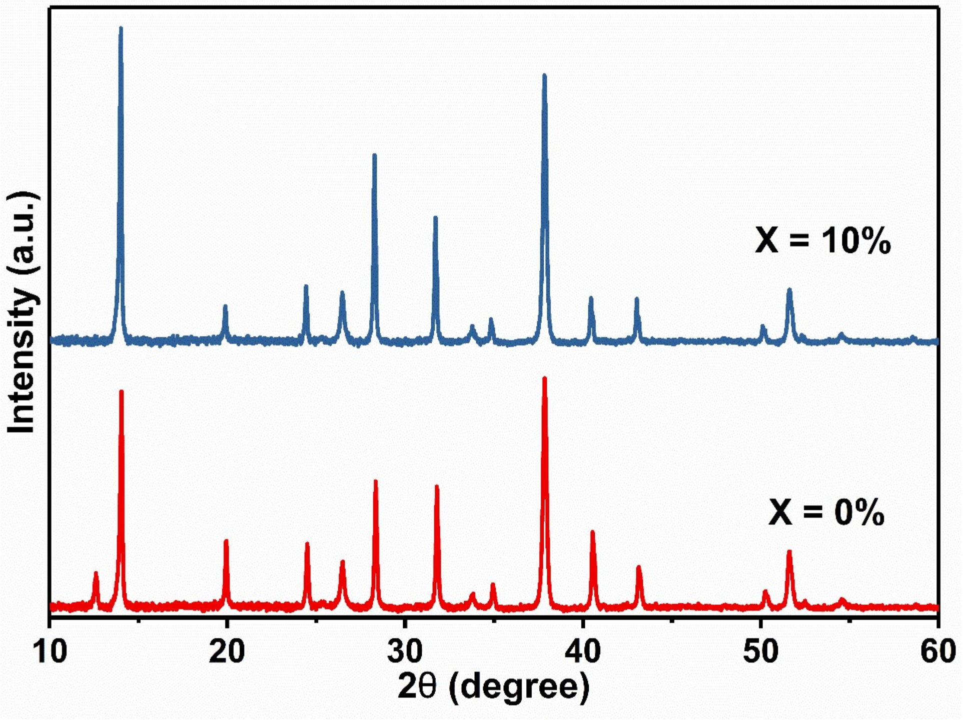

Fig S10 XRD patterns of triple-cation perovskite films cast on TiO_2 substrates from precursor solutions having initial KI concentrations of 0 and 10%.

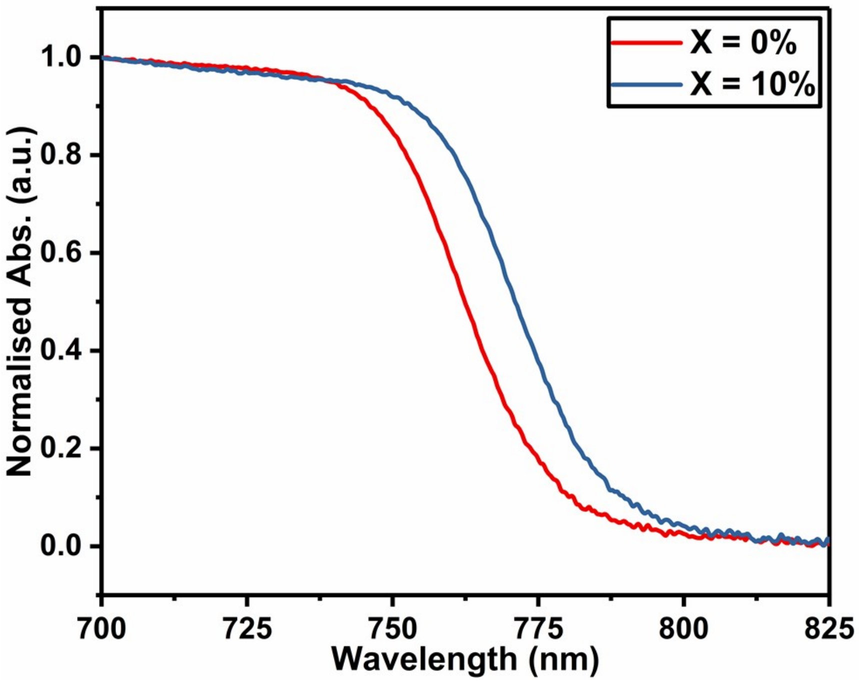

Fig S11 UV-Vis absorption spectra of triple-cation perovskite films cast on c-TiO_2/m-TiO_2 substrates from a precursor solution having initial KI concentrations of 0 and 10%.

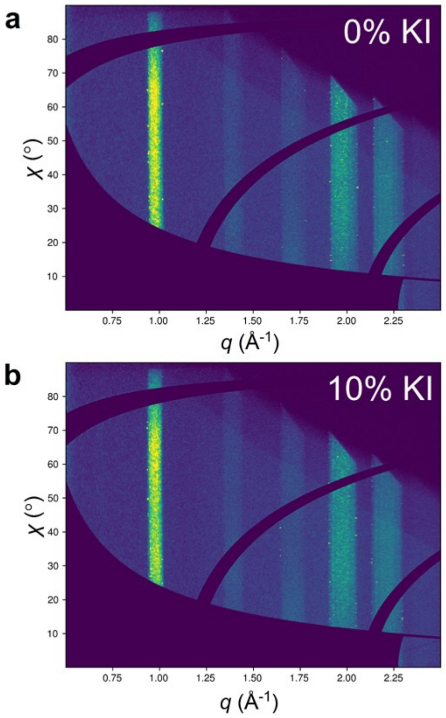

Fig S12 Typical remapped 2D scattering patterns for the 0% KI and 10% KI samples as recorded prior to accelerated aging conditions. Data are plotted as χ (azimuthal scattering angle) vs. q (momentum transfer) with the perovskite (100) reflection observed at q ≈ 1.0 $Å^{-1}$. For in-situ degradation studies, integrated scatter from this reflection was monitored, with background subtraction.

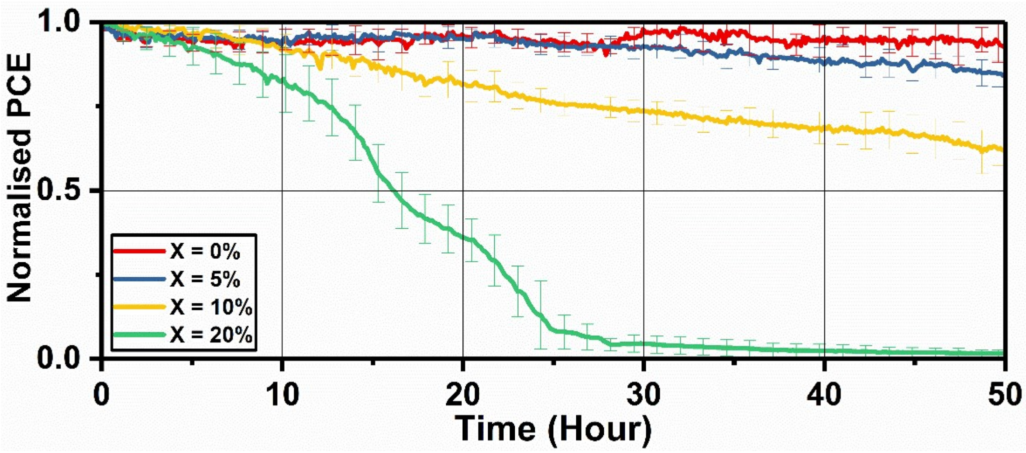

Fig S13 Stability of unencapsulated perovskite devices with different concentration of KI added to the precursor solution. Curves were constructed from data taken from a minimum of 2 devices.

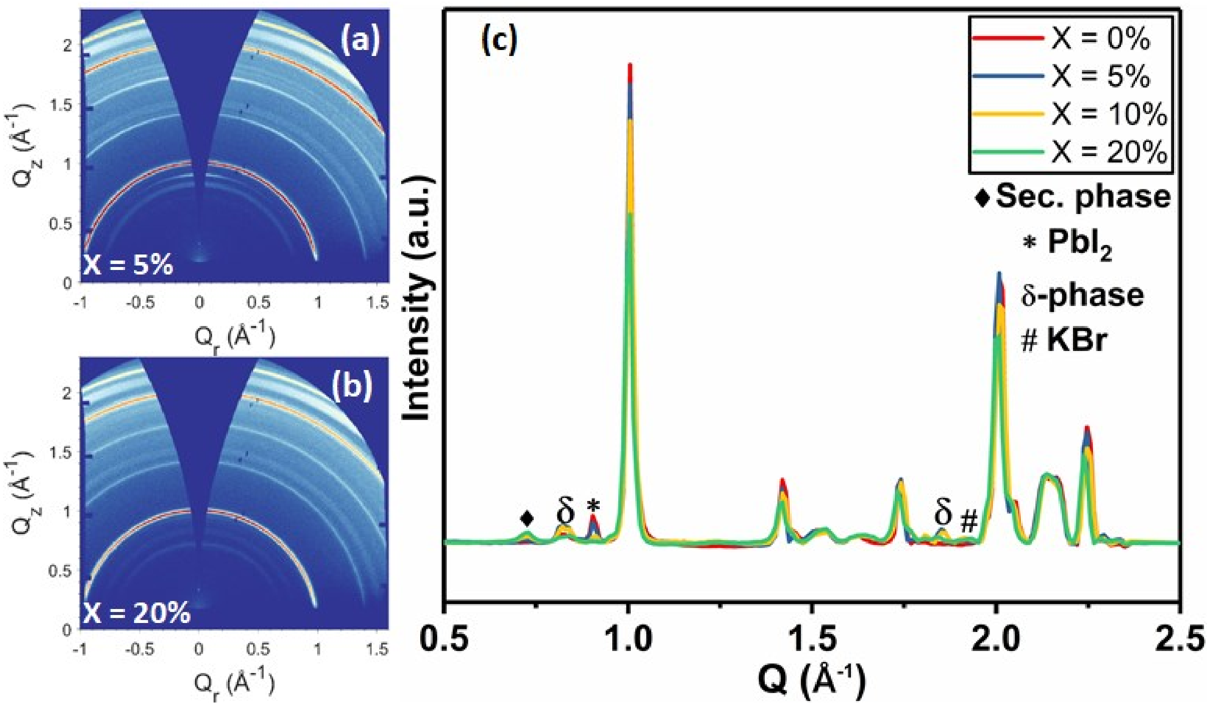

Fig S14 GIWAXS of films taken from aged devices, with the perovskite films prepared from precursors containing KI at a concentration of (a) X = 5% and (b) X= 20%. Part (c) shows an azimuthal integration of the GIWAXS pattern from an aged device recorded over profile $0.5 \leq Q \leq 2.5$ Å^{-1}.

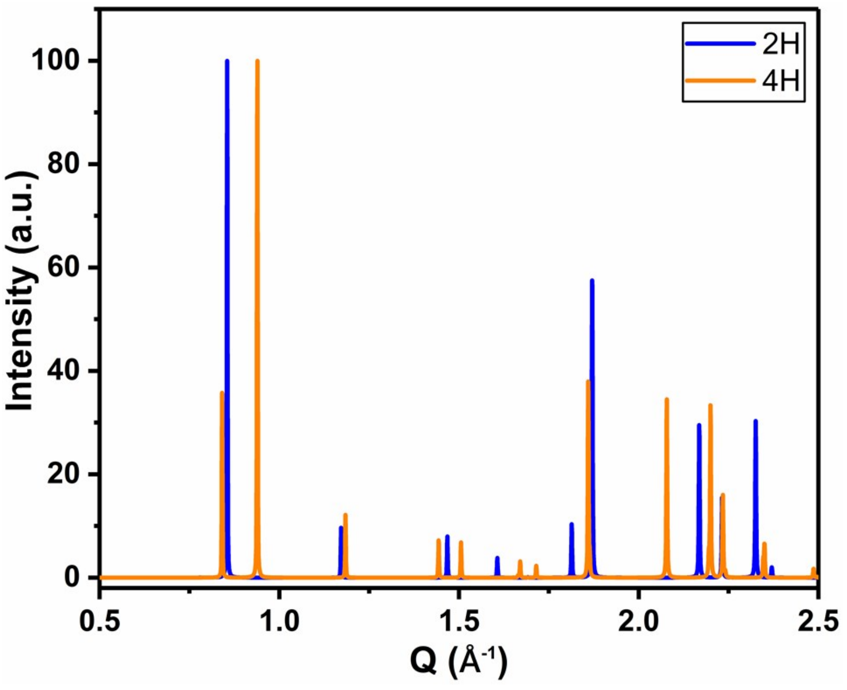

Fig S15 Simulated XRD of 2H and 4H polytypes of $FAPbI_3$.

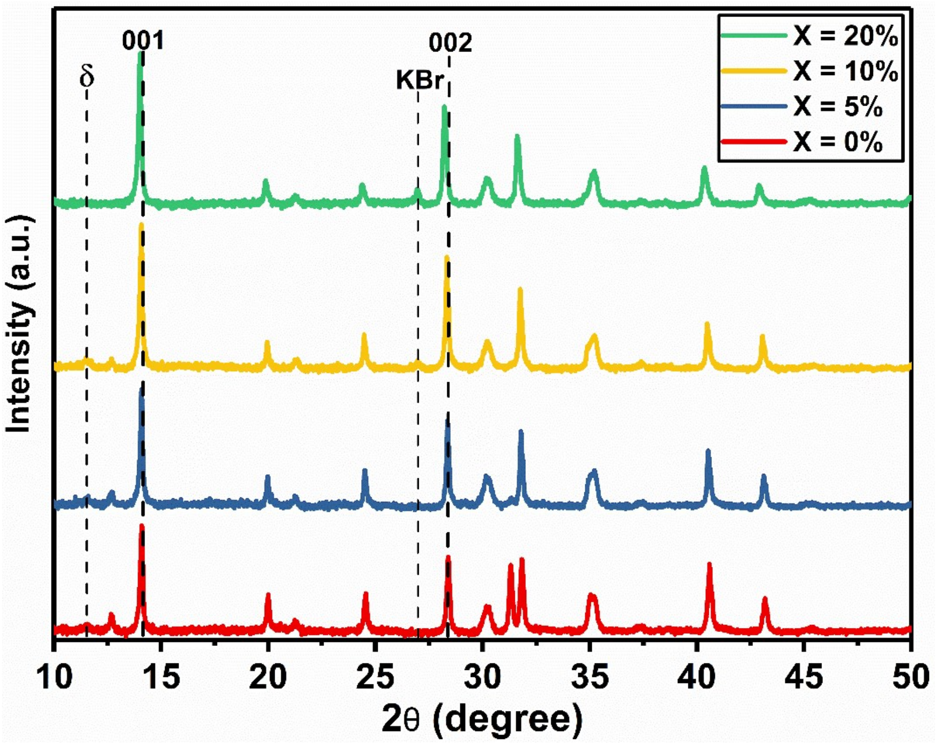

Fig S16 XRD patterns of aged triple-cation perovskite devices containing different concentrations of KI added to the precursor.

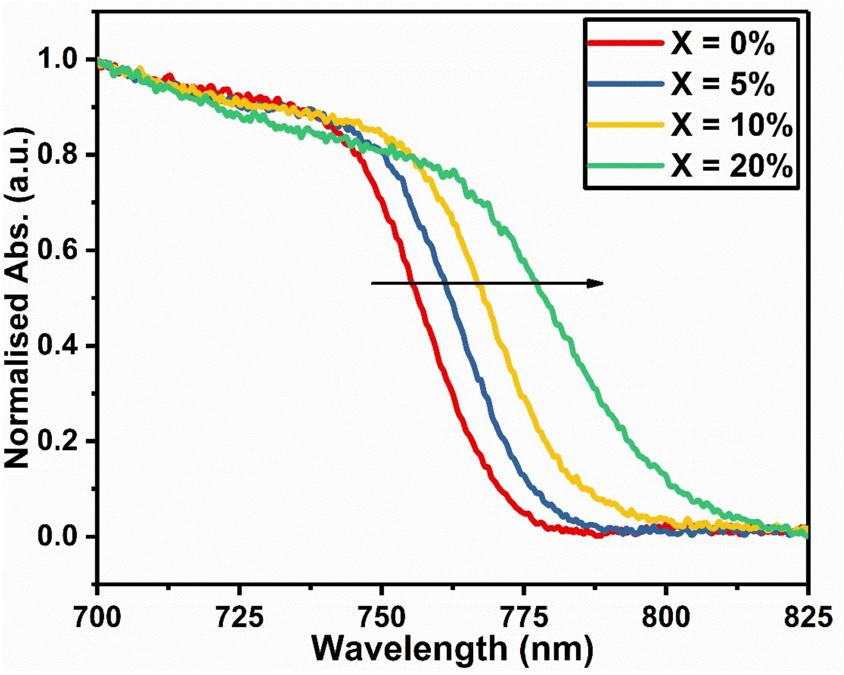

Fig S17 Normalised UV-Vis absorption for aged triple-cation perovskite films with X =0, 5, 10 and 20%.

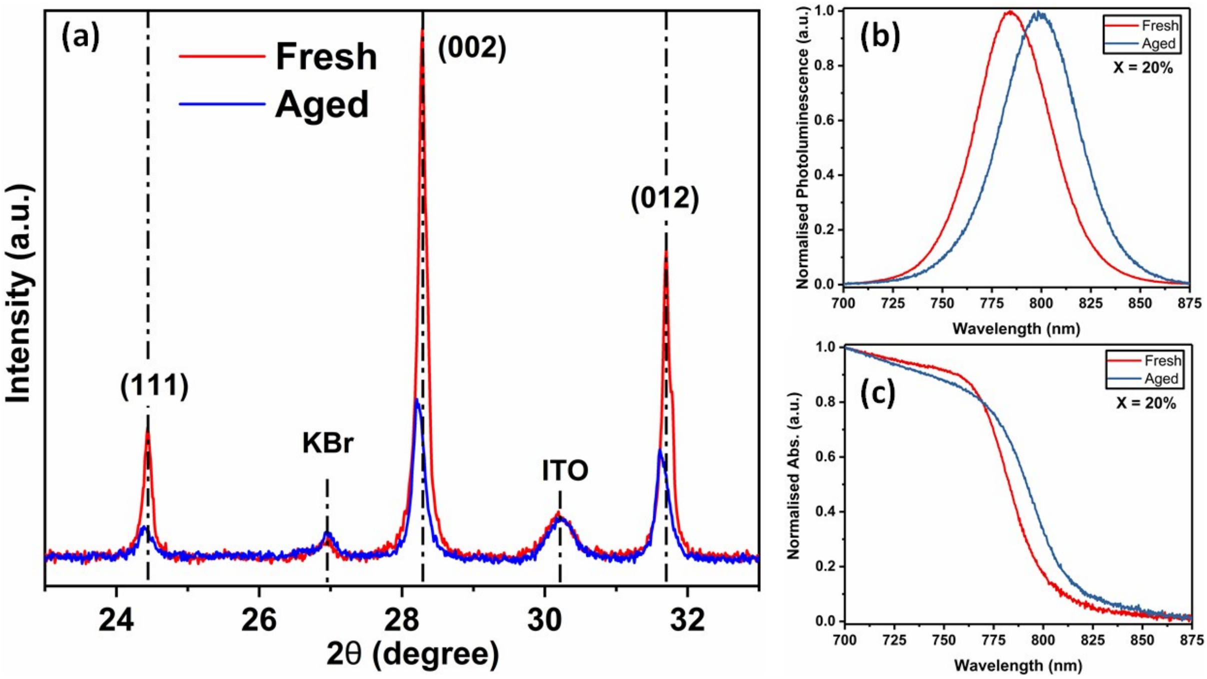

Fig S18 (a) XRD patterns, (b) Normalised UV-Vis absorption and (c) Normalised photoluminescence recorded on freshly prepared and aged TC perovskite films with X= 20%.

References

1 Z. Jiang, *J. Appl. Crystallogr.*, 2015, **48**, 917–926.

2 O. S. Game, J. A. Smith, T. I. Alanazi, M. Wong-Stringer, V. Kumar, C. Rodenburg, N. J. Terrill and D. G. Lidzey, *J. Mater. Chem. A*, 2020, **8**, 10943–10956.

3 G. Ashiotis, A. Deschildre, Z. Nawaz, J. P. Wright, D. Karkoulis, F. E. Picca and J. Kieffer, *J. Appl. Crystallogr.*, 2015, **48**, 510–519.

4 S. N. Habisreutinger, N. K. Noel and H. J. Snaith, *ACS Energy Lett.*, 2018, **3**, 2472–2476.
